# The Association Between HIV Infection and Carotid Intima-Media Thickness in the Era of Antiretroviral Therapy: A Meta-Analysis

**DOI:** 10.3390/v17070894

**Published:** 2025-06-25

**Authors:** Angelina Nieuwoudt, Kay-Lee E. Strauss, Wendy N. Phoswa, Kabelo Mokgalaboni

**Affiliations:** Department of Life and Consumer Sciences, College of Agriculture and Environmental Sciences, University of South Africa, Roodepoort 1710, South Africa; 67184464@mylife.unisa.ac.za (A.N.);

**Keywords:** antiretroviral therapy, atherosclerosis, carotid intima-media thickness, cardiovascular risk, human immunodeficiency virus

## Abstract

Atherosclerosis remains a leading cause of mortality globally, and this is worse in people living with HIV (PLHIV). While the administration of antiretroviral therapy (ART) in this population has significant benefits, it is essential to acknowledge that it also has some undesired effects. This study investigated the impact of ART on carotid intima-media thickness (CIMT) in PLHIV as a marker of early atherosclerosis. A literature search was conducted on the PubMed, Scopus, and EBSCOhost databases from 1 January 1987 to 30 May 2025. The methodological quality of the studies was assessed using the Newcastle–Ottawa scale. Data were analyzed using a meta-analysis web tool and reported as the mean difference (MD) and 95% confidence intervals (CIs). Twenty-seven studies, which included 3250 PLHIV on ART and 1542 who were ART-naive, were relevant. The mean age was 41.26 in ART and 39.91 years. The results showed a higher CIMT in PLHIV on ART compared to the ART-naive group, MD = 0.03 mm, 95% CI (0.02 mm to 0.04 mm), *p* < 0.0001; I^2^ = 96.9%. Subgroup analysis showed that the inclusion of studies conducted on male participants only, those with a sample size of one hundred, and those with a moderate risk of bias contributed to heterogeneity. The results suggest there is an increased risk of atherosclerosis in PLHIV on ART.

## 1. Introduction

Human immunodeficiency virus (HIV) causes an infection that targets the white blood cells to weaken the immune system, causing individuals to be more vulnerable to various infections, including cardiovascular diseases (CVDs) [1]. According to the World Organization (WHO) HIV report of 2023, there were 39.9 million people living with HIV (PLHIV) worldwide [2]. South Africa (SA) has a considerable proportion of the global HIV. It was estimated that there were approximately 8.45 million PLHIV in SA in 2022 [3]. Therefore, SA is recognized as the country with the highest number of PLHIV worldwide. This suggests that the use of antiretroviral therapy (ART) and the prevalence of CVD in SA may rise in this population. The effective use of ART has become a crucial strategy in preventing HIV-associated infections and CVD amongst PLHIV. It has resulted in a significant decrease in mortality and mortality rates among PLHIV [4,5].

However, despite ART’s success, existing research has revealed the association between HIV infection, ART, and the risk of CVDs [6]. Furthermore, ART is associated with metabolic disturbances like insulin resistance, dyslipidemia, and lipodystrophy syndrome [7]. Recent evidence suggests HIV and ART are independently related to CVD development [1]. Strauss and colleagues recently reported a significant decrease in vascular adhesion molecules in PLHIV on ART, suggesting improved endothelial function. However, the same study demonstrated inconsistent results regarding flow-mediated dilation [8]. Therefore, these raise conflicting conclusions about the effect of ART on CVD development. While the impact of ARTs on the overall health of PLHIV is well documented, their contribution to the pathogenesis of CVDs is not widely understood, due to the synergistic effects of HIV infection and ART on CVDs [9]. Other studies have shown that specific forms of ART regimens are associated with a high risk of CVDs. Additionally, exposure to protease inhibitors (PI) is associated with an increased risk of myocardial infarction [10]. Another study revealed that exposure to abacavir is associated with a high risk of CVDs [11].

Among these CVDs, atherosclerosis is more prevalent among PLHIV regardless of ART [12,13]. Atherosclerosis is characterized by the buildup of plaque in the arteries, impairing the normal functioning of blood vessels [14]. Among PLHIV, atherosclerosis is more prevalent and a significant health challenge, thus warranting a need to curb the associated complications. Carotid intima-media thickness (CIMT) is an ideal noninvasive surrogate marker of subclinical atherosclerosis and is used clinically to identify the risk of cardiovascular events among PLHIV early [15]. High CIMT values indicate an increased risk of CVDs. In PLHIV, a high CIMT could suggest accelerated atherosclerosis due to HIV infection-related factors such as chronic inflammation, immune activation, and ART exposure [16]. Recently, Vos and colleagues reported a significant increase in CIMT among PLHIV [17], further confirming that PLHIV are at high risk of CVDs. Therefore, assessing this marker may be beneficial in controlling the rising rate of CVD among PLHIV.

Although PLHIV exhibit a higher predisposition to atherosclerosis [18,19], the overall effect of ART on CIMT is poorly understood, as some studies have shown a higher CIMT [20,21] and others have shown no effect at all [22,23,24] irrespective of ART use among PLHIV. More recently, other researchers have reported through meta-analysis the contribution of ART to CIMT progression in HIV children and those infected [25]. Another group also explored the contribution of ART to CIMT in PLHIV compared to those who were HIV-negative [26]. However, there has been no meta-analysis of the CIMT progression among PLHIV and those who are ART-naive. As these two groups are positive, the CIMT progression might also be attributed to HIV infections. Therefore, this study aimed to examine the overall impact of ART on CIMT in adult PLHIV and those who are ART-naive.

## 2. Materials and Methods

### 2.1. Literature Search Strategy

The study adhered to the Preferred Reporting Items for Systematic Review and Meta-Analysis (PRISMA) guidelines [27]. The study was not registered with any registry bodies; however, the Cochrane Library and PROSPERO were searched to avoid duplicating a similar study. To investigate the relationship between CIMT and atherosclerosis in PLHIV using ART, two investigators (A.N. and K.M.) independently conducted a comprehensive literature search on PubMed, Scopus, and EBSCOhost. The search utilized keywords and Boolean operators (AND, OR) to ensure a thorough exploration of the relevant literature. The search terms included “Antiretroviral Therapy”, “combined antiretroviral therapy”, “HIV”, and “Carotid Intima-Media Thickness”, combined using “OR” and AND operators to filter and refine the results. The search strategy was refined by applying several filters to enhance the relevance and quality of the studies. We restricted our search to publications in English to ensure accurate interpretation and analysis (Table S1). The search was restricted to records published from 1 January 1987, to 15 October 2024, and further updated on 30 May 2025. This was partly based on the timeline by which the WHO approved the first ART regimen [28]. Additionally, we reviewed the reference lists of selected papers to identify further relevant studies. The level of agreement was assessed using an online Kappa (K) calculator: http://justusrandolph.net/kappa/ (accessed on 14 November 2024). A Kappa (K) value was used to classify the agreement based on established guidelines [29].

### 2.2. Inclusion and Exclusion Criteria

To ensure the relevance and quality of the studies in this review, we adhered to specific guidelines outlined in the PICOS criteria. These were defined as follows: population (P) was adults aged 18 years and older, regardless of gender, who were living with HIV. The intervention (I) was any form of ART, including HAART or combined ART regimens. Furthermore, a control (C) group included PLHIV who were ART-naive, while the outcome (O) was CIMT status. The study design (S) included the following: cohorts, case–control, and cross-sectional studies.

We excluded studies without an ART-naive group that did not measure CIMT. To ensure accuracy in interpretation, we restricted our review to studies published in English. Studies on non-HIV populations, those without an ART-naive group, those involving pediatric participants, and those published as reviews, meta-analyses, conference abstracts, letters, preclinical studies, protocols, and non-peer-reviewed grey literature (including theses, preprints, and dissertations) were excluded.

### 2.3. Study Selection Process

The process of selecting studies was performed by three independent investigators (A.N., K.E.S., and K.M.), which involved three stages: screening titles and abstracts, identifying duplicate records, and screening full texts to ensure they adhered to the predefined eligibility criteria. This screening process was critical to filter out studies that did not meet our predefined requirements. The final selection of studies was guided by the PICOS framework, focusing on adults diagnosed with HIV, with interventions involving HAART, CART, or other forms of ART, and a control group (PLHIV who are ART-naive). The outcome was CIMT, which is an indicator of atherosclerosis.

### 2.4. Data Extraction and Management

Two investigators (N.A., K.E.S.) independently conducted the data extraction process to reduce bias and ensure data accuracy. A predefined Microsoft 365 Excel sheet was used to record key information from each study. The main items extracted from each study included the first author’s last name, country of publication, study design, population characteristics, participants’ age and BMI, CD4 count in both groups, duration since HIV diagnosis, CIMT values in both groups, the number and percentage of male participants, and a summary of the findings. After completing the initial extraction, the data from each investigator was compared by an independent investigator (K.M.) to assess any disagreement. In cases where discrepancies were found, these were resolved by K.M. through re-evaluation of the items or studies in question and sharing the final decision with the other investigators. This feedback was established to enhance accuracy and transparency, ensuring that all aspects of study quality are thoroughly examined. The Mendeley reference manager (version 2.119.0) was used to save all retrieved studies and identify duplicates.

### 2.5. Methodological Quality Assessment and Risk of Bias

The Newcastle–Ottawa scale (NOS) rating scale was used to evaluate the quality of cohorts, case–control, and cross-sectional studies [30]. This scale assesses the quality of studies across three primary domains: the selection of study groups, the comparability of groups, and the evaluation of outcomes or exposure. Each domain was scored to determine the study’s overall quality and risk of bias. The study was regarded as high, moderate, or low based on the scores. A score of 7–9 was high, 5–6 was moderate, and those below 4 were of low quality. Studies were classified as having a low risk of bias if they had high quality and a moderate risk of bias if they had moderate quality. This assessment was independently conducted by A.N. and W.N.P.; where there was disagreement in rating scores, K.M. conducted an independent evaluation to conclude the rating and quality of the study and the domain in question.

### 2.6. Data Preparation and Analysis

Microsoft Excel (version 2503) assisted in the preliminary data organization, extraction, and basic calculations. The data computed included the mean, standard deviation (SD), and sample size for each study group. For studies reporting the median and range, mean and SD values were estimated according to established guidelines [31]. When the standard error of the mean (SEM) was reported, SD was derived using the formula SD = SEM × √n. Studies with multiple ART groups were combined sequentially into a single group (treatment group) using the Cochrane formula (https://www.statstodo.com/CombineMeansSDs.php, accessed on 23 November 2024). Data reported as micrometers were converted to millimeters before computation. The mean difference (MD) was used to present the effect estimates for CIMT with 95% confidence intervals (CI) reported. Statistical heterogeneity was assessed using the I^2^ statistic, with thresholds indicating low heterogeneity (<50%) and substantial heterogeneity (≥75%) [32]. The I^2^ value will be reported to reflect the extent of heterogeneity observed. Funnel plots and Egger’s test were used to evaluate publication bias [33,34]. Subgroup analysis was used to investigate the source of heterogeneity [35]. This was performed based on gender (male, female, or studies that included both), age (below an above 40), sample size (below, above, or equal to 100), continent (Africa, Asia, or Europe), study design, and quality/risk of bias of the study (high, low, and moderate). A subgroup analysis based on the duration of ART initiation was also conducted, following the WHO recommendation for rapid ART initiation within 7 days of HIV diagnosis (subgroups for studies conducted before 2015 and those conducted after 2015). This was done by examining the year the study was conducted, rather than when it was published. Furthermore, a subgroup based on duration since HIV diagnosis was performed. This was categorized into short, medium, and long duration. Subgroup analysis based on the CD4 count of both groups was also conducted. This was classified as less than 200 cells/mm^3^, 200 to 349 cells/mm^3^, 350 to 499 cells/mm^3^, and ≥500 cells/mm^3^. Sensitivity analysis was performed to test the robustness of the results. This was performed by excluding one study at a time and re-analyzing the effect size [36]. A freely accessible meta-analysis web tool was used for data analysis [37]. The results were presented using a forest plot as MD and 95% CI. A two-tailed test was used, with statistical significance set at *p*-values < 0.05.

## 3. Results

### 3.1. Overview of Search and Selection

**Figure 1:**
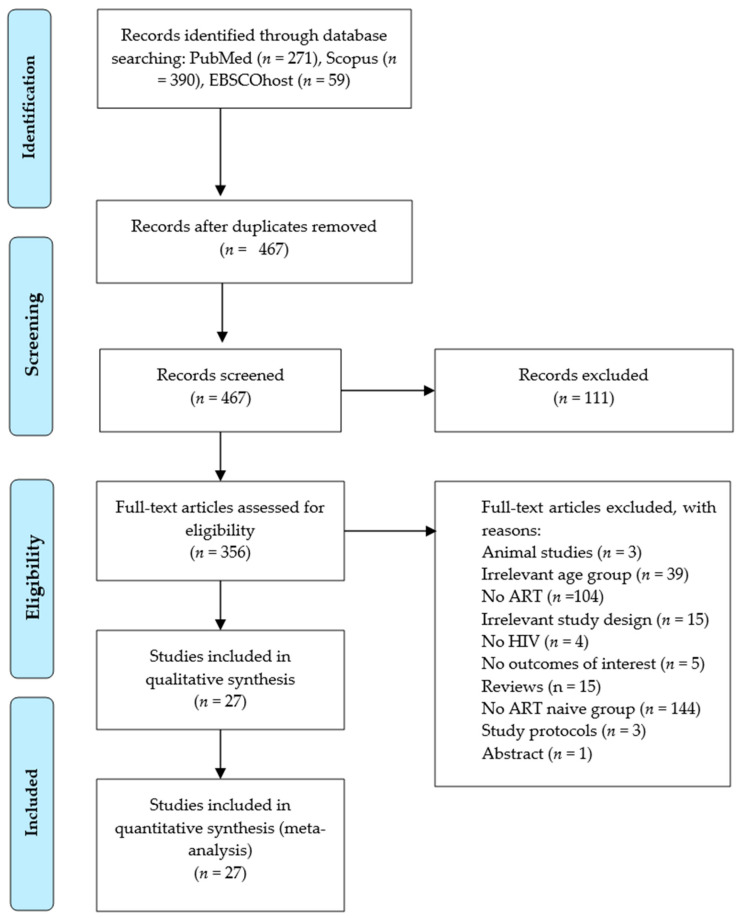
Preferred Items for Systematic Review and Meta-Analysis (PRISMA) flow diagram.

The identification of studies was made from PubMed (*n* = 271), Scopus (*n* = 390), and EBSCOhost (*n* = 59). Following the initial screening, 253 duplicates were removed. Then, the remaining 467 records were screened based on their titles and abstracts, resulting in the exclusion of 111 records due to irrelevant titles and abstracts, and 3 studies on animal models. Of those records subjected to full screening, 104 were excluded because they did not involve an ART intervention, 39 studies were excluded due to irrelevant age groups (children and adolescents), and 15 were excluded as review articles. Further exclusions included 21 studies with irrelevant study designs as per our PICOS. Additionally, 144 studies lacked a control group (ART-naive), while 5 studies showed no CIMT as an outcome of interest. Lastly, one conference abstract and three protocols were also excluded. After these exclusions, 27 studies [20,21,24,38,39,40,41,42,43,44,45,46,47,48,49,50,51,52,53,54,55,56,57,58,59,60,61] satisfied the preplanned PICOS criteria and were included in the meta-analysis (Figure 1).

### 3.2. Basic Characteristics of Included Studies

Table 1 provides an overview of the general characteristics of the studies included in this meta-analysis. Twenty-seven studies [20,21,24,38,39,40,41,42,43,44,45,46,47,48,49,50,51,52,53,54,55,56,57,58,59,60,61] met the inclusion criteria. The sample size consisted of 4792 participants, including 3250 individuals on ART and 1542 who were ART-naive. PLHIV in the ART groups showed a wide range of characteristics. The number of male participants in the group on ART in the 22 studies was 1054 (33.4%). In the ART-naive group, there were 503 (33.7%) male participants from 20 studies. The mean age reported in the ART group was 41.26 ± 7.73 years, and in the ART-naive group, it was 39.91 ± 11.24 years. The mean BMI in the ART group was 26.5 ± 5.0 kg/m^2^ and 24.46 ± 7.31 kg/m^2^ in the ART-naive group.

The studies were conducted across a range of countries. South Africa had the largest number of studies [21,24,41,51,53], followed by Italy [46,55,58,59] and India [20,44,57,60,61]. The United States of America contributed two studies [45,56]. Additionally, a single study was conducted in each of the following: Botswana [38], Colombia [39], China [43], Ethiopia [52], France [42], Ghana [49], the Netherlands [47], Russia [40], and Spain [48]. One study was conducted jointly in Kenya and South Africa [50]. Different research designs were employed across these studies. Twenty-two studies used a cross-sectional design [20,21,24,38,39,40,42,43,44,45,46,49,50,51,52,53,54,55,58,59,60,61], two case–control [47,57], and three studies [41,48,56] were designed as cohorts (retrospective and prospective).

### 3.3. The Effect of ART on Carotid Progression in PLHIV

**Figure 2:**
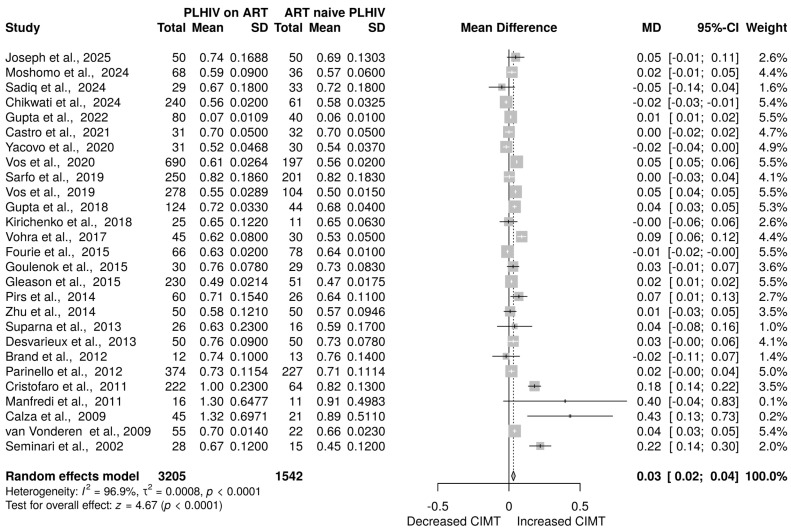
CIMT in PLHIV on ART versus ART-naive PLHIV. Data are reported as the mean difference and 95% confidence intervals [20,21,24,38,39,40,41,42,43,44,45,46,47,48,49,50,51,52,53,54,55,56,57,58,59,60,61]. The gray box shows the weight of the study, the solid vertical line shows the line of no effect, the dashed vertical line shows the effect size, the horizontal line shows the confidence intervals, diamond plot shows the overall effect estimates.

Twenty-seven studies [20,21,24,38,39,40,41,42,43,44,45,46,47,48,49,50,51,52,53,54,55,56,58,59,60,61] with a sample size of 4792 (3205 on ART and 1542 ART-naive) was examined to assess the impact of ART on CIMT. The overall effect size from the random-effects model meta-analysis indicated a significant increase in the level of CIMT in HIV-positive patients on ART compared to those who were ART-naive [MD = 0.03 mm, 95% CI (0.02 mm to 0.04 mm), *p* < 0.0001] (Figure 2). However, there was a high level of heterogeneity among the included studies, with I^2^ = 96.9% (*p* < 0.0001) (Figure 2).

### 3.4. Assessment of Publication Bias

**Figure 3:**
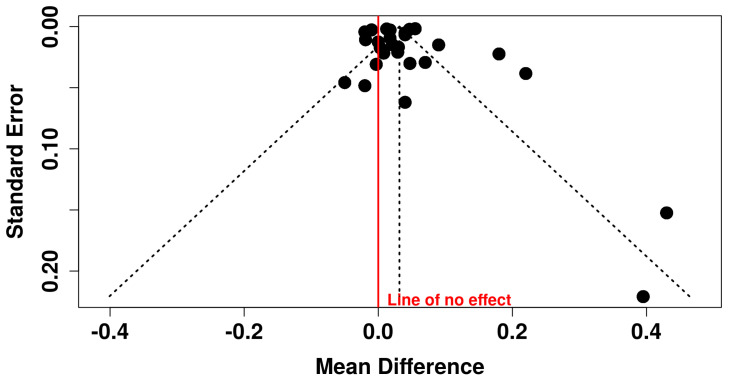
Funnel plot showing assessment of publication bias.

Publication bias was assessed using visual inspection of funnel plots and statistically through the Egger test. Notably, no evidence of potential publication bias was observed through visual inspection of the funnel plot (Figure 3). This was confirmed by Egger’s test (intercept: −0.05 mm, 95% CI: −2.91 mm to 2.81 mm, t: −0.034, *p* = 0.973).

### 3.5. Subgroup Analysis

As a result of high heterogeneity among the studies (I^2^ = 97%) (Figure 2), a subgroup analysis was conducted. The evidence published in America seems to have contributed to the heterogeneity, as subgroup analysis showed minimal heterogeneity (I^2^ = 11.9%) (Figure S1A). A CD4 count below 500 introduced minimal heterogeneity (Figure S1B). The inclusion of studies conducted on male participants was found to be one factor contributing to statistical heterogeneity, as subgroup results revealed zero heterogeneity (I^2^ = 0%) compared to those that included either females or both genders (Figure S1C). Cohorts minimally contributed to heterogeneity (Figure S1D). Studies with a sample size of 100 were also deemed as contributors of heterogeneity (Figure S1E). However, the age of participants led to minimal heterogeneity (Figure S1F). The quality of studies was also deemed as a contributing factor, as those with moderate quality/ROB were associated with heterogeneity (Figure S1G). The evidence also showed that the duration of publication might have contributed to heterogeneity, as studies published before the WHO adopted rapid ART initiation showed a minimal reduction in heterogeneity after subgroup analysis (Figure S1H). Interestingly, the subgroup showed that the duration of HIV infection was a contributing factor, as a long duration (greater than 10 years) of HIV infection was associated with reduced heterogeneity post-subgroup (Figure S1I). However, when studies were subgrouped based on the duration of HIV infection since diagnosis in ART-naive individuals, there was no change in heterogeneity (Figure S1J).

### 3.6. Sensitivity Analysis

To perform a sensitivity analysis for each study’s effect on the effect size of this dataset, one study was removed at a time. The results of the sensitivity analysis are shown in Table S2. Notably, after excluding most of the above-mentioned studies, there was no significant change in the effect size. However, the exclusion of Cristofaro et al., 2011 [46] and Seminari et al. [58] led to a slight decrease in effect size, 0.01 mm, 95%CI (0.01 mm to 0.04 mm).

### 3.7. Quality Assessment

The overall quality and risk of bias across all studies are presented in Supplementary Tables S3–S5. In the quality assessment of cohort studies using the NOS, three studies were rated as high quality with total scores of 7–8 [41,48,56] (Table S3). For the case–control studies, two scored between 7 and 8 stars and thus were classified as high quality [47,57] (Table S4). Among the cross-sectional studies, nineteen studies [21,24,38,40,42,43,45,46,49,50,51,52,53,54,55,58,59,60,61] received high ratings, with scores ranging from 7 to 8, thus suggesting good quality (Table S5). An additional three cross-sectional studies scored six ratings, indicating moderate quality [20,44,60] (Table S5).

## 4. Discussion

Atherosclerosis is a vascular complication, and this can lead to severe adversity in the HIV-positive population. The CIMT has emerged as a noninvasive marker for subclinical atherosclerosis, and it is elevated in PLHIV, indicating early signs of vascular damage [6,62]. While PLHIV rely on ART to suppress the viral load and improve the immune system, adverse cardiovascular, hepatic, and renal events are also reported [63,64]. This meta-analysis comprised 27 studies evaluating the effect of ART on CIMT in PLHIV. The results revealed a significantly higher CIMT level in PLHIV on ART than in ART-naive individuals. The findings from subgroup analysis indicated that the inclusion of studies on male participants, those with moderate quality, and those with sample sizes of 100 were the significant contributing factors to the heterogeneity. Following the subgroup analysis, the studies published in America revealed a low heterogeneity (I^2^ = 12%), suggesting that geographical location may be a contributor to the observed variation. This result can be partially attributed to regional differences in access to health resources, ART medication, and information about HIV and ART.

The observed increase in CIMT in PLHIV on ART can be explained by different mechanisms associated with either HIV infections or ART. HIV infection results in persistent inflammation even after ART treatment due to residual viral replication and the production of viral proteins that contribute to immune activation, thus creating an environment conducive to the development of atherosclerosis [65,66]. This persistent inflammation is characterized by an increased production of proinflammatory cytokines, which contribute to endothelial dysfunction, therefore promoting the thickening of the CIMT [67,68]. Increased levels of proinflammatory cytokines promote vascular damage, resulting in a progressive increase in CIMT over time [4]. Moreover, HIV-infected monocytes and macrophages remain chronically activated, contributing to inflammation and plaque formation in the arteries [66]. The viral proteins, such as negative regulatory factors (Nefs), can exacerbate oxidative stress, thereby further damaging endothelial cells [69]. These results highlight the contribution of chronic inflammation and vascular damage to the progression of CIMT. ART regimens, especially protease inhibitors (PIs), are associated with insulin resistance and abdominal obesity, which can impair lipid metabolism [70,71,72]. It is worth noting that insulin resistance promotes a proinflammatory and prothrombotic status; collectively, these factors contribute to atherosclerosis and CIMT progression [65,73]. Dyslipidemia, as characterized by the accumulation of lipids (increased cholesterol and triglycerides) in the arterial wall, forms plaques that increase arterial stiffness and CIMT progression [74,75]. ART, as it restores immune function, fails to normalize it, resulting in persistent immune activation, which transforms macrophages into foam cells, thus contributing to the formation of atherosclerotic plaques [4]. Moreover, other ART regimens, especially combined therapy, directly promote oxidative stress, resulting in endothelial dysfunction and foam cell formation in macrophages [76,77]. This process is exacerbated by viral proteins that promote reactive oxygen species (ROS) production [69,78]. The initiation of the ART regimens, especially the older regimens, is associated with lipodystrophy, characterized by abnormal fat distribution and visceral adiposity [79]. These findings suggest that exposure to ART may affect endothelial function through modifications to the lipid profile induced by oxidative stress [79,80]. Altogether, these contribute to persistent vascular inflammation and CIMT progression.

Compared to findings from other studies, this meta-analysis supports recent evidence by [24,81], whereby participants treated with PI-based ART regimens had a significantly higher CIMT. ART-associated metabolic alterations, endothelial dysfunction, dyslipidemia, and insulin resistance have been reported to be associated with vascular dysfunction, especially in elderly patients [54,82,83]. Majonga and team [25] also showed an increased CIMT; however, this study was conducted in children living with HIV exposed to ART. Moreover, this study did not have an ART-naive group, and the study was underpowered as evidence from seven studies was quantitatively synthesized. Therefore, the results might not necessarily account for the effect of HIV infection on the thickness of the arteries. On the contrary, other researchers have reported the cardioprotective effect of ART, as shown by reduced CIMT post-ART compared to ART-naïve patients [48,50]. For instance, a recent study by Chikwati et al. (2024) reported a substantial decrease in CIMT among PLHIV from South Africa compared to ART-naive individuals [50]. However, it is important to note that while it was recently published, this study was conducted between 2013 and 2016, the period during which the national HIV guide was recommending efavirenz-based regimens. Moreover, this study was skewed in terms of sample size and population; female participants were recruited, and more participants were in the ART (240) compared to the ART-naïve group (61). The same study failed to report information about the duration of HIV infection, ART use, and CD4 counts. Similarly, a study conducted in between 2012 and 2013 among ART-exposed PLHIV in Spain consistently showed a lower CIMT among ART-exposed patients, which may be due to its reduced metabolic side effects and low vascular inflammation [48]. The differences between these findings and the current meta-analysis highlight a complex relationship between HIV and ART and their contribution to vascular impairment. Other studies have compared CIMT status among PLHIV on ART and those not treated; their findings suggest that the impact of ART on vascular health may depend on external factors, such as diet, exercise, and genetics [84,85]. Newer ART regimens, especially dolutegravir, have demonstrated low rates of metabolic side effects and a positive impact on carotid progression compared to PIs [86]. Evidence from other studies suggests that ART has other benefits beyond the viral load reduction [8,16,48]. This includes improvements in endothelial function, reduced inflammation, and CIMT progression [8,16,48]. These results suggest that specific ART regimens, such as dolutegravir and efavirenz-containing ART regimens, are more effective and may have a significant impact on vascular health [24,86]. All together, these may contribute to improved vascular health.

Several strengths and limitations must be highlighted and considered when interpreting the results of this study. The study is reported in accordance with PRISMA (Table S6). The main limitation is that the included studies failed to identify the type of ART regimen used, as some studies did not specify the form of ART used. However, our subgroup based on the WHO adoption of rapid ART initiation in 2015/2016 clarified the impact of this approach and also, to some extent, might have given us an idea of which form of ART was used based on the duration. In addition, the age and BMI of participants were not reported in other studies, limiting our overall interpretation of BMI and its influence on ART therapy and endothelial dysfunction. Although substantial heterogeneity was observed, a subgroup analysis was conducted to identify the sources, which were deemed to be the inclusion of studies conducted on male participants only, those with a sample size of 100, and those published in the United States. Sensitivity analysis was also performed, and no significant changes in the effect size were noted. The comprehensive search on multiple databases ensured that all relevant studies were identified. The search, screening, and extraction processes were also independently performed to avoid bias and ensure reliability. The analysis had a large and diverse sample size from twenty-seven studies, with 3250 PLHIV on ART and 1542 who were ART-naive. The large sample size enhanced the statistical power of the results, improving accuracy and reliability. By incorporating older and more recent studies from 2002 to 2025, the meta-analysis provided valuable insights into the change in CIMT values over time. It reflected changes in research focus, methodologies, and the application of ART. Of the evidence analyzed in this study, 89% was classified as high and 11% as moderate quality according to the Newcastle–Ottawa scale. While a subgroup based on duration of HIV infection was performed, unfortunately, only 13 (48%) studies reported the duration since HIV infection in both groups.

## 5. Conclusions

This study found that PLHIV on ART had significantly higher CIMT values than the ART-naive group. This suggests that ART administration among this population is associated with CIMT progression, thus predisposing this population to the development of atherosclerosis. An increased CIMT value indicates the early stages of atherosclerosis and can serve as an early marker of the disease. Therefore, these results suggest that regular CIMT monitoring among PLHIV on ART is important to prevent atherosclerosis and associated CVD. Thus, more highly powered clinical trials should be conducted to establish the association between ART and CIMT progression.

## Figures and Tables

**Table 1:** General characteristics of included studies.

Authors, Year of Publication	Countries	When the Study Was Conducted	Design	Population Size	Duration Since HIV Diagnosis (Years)	Male. N (%)	Age (Years) Mean ± SD	BMI (kg/m ^2^) Mean ± SD	CD4 Count Mean and SD	Key Findings	ROB
Joseph et al., 2025 [61]	India	NR	Comparative cross-sectional	PLHIV on ART (50) PLHIV on ART (50)	NR	NR	NR	NR	NR	No significant difference in CIMT between the groups	Low
Chikwati et al., 2024 [50]	Kenya, South Africa	2013 and 2016	Cross-sectional	PLHIV on ART (240) ART-naive (61)	NR	ART: 0 (0) ART-naive: 0 (0)	ART: 47.6 ± 5.5 ART-naive: 47.5 ± 6.0	ART: 26.1 ± 6.3 ART-naive: 30.8 ± 8.3	NR	Significant decrease in CIMT in ART compared to ART-naive patients	Low
Sadiq et al., 2024 [24]	South Africa	2014 to 2017	Cross-sectional	ART (29) ART-naive (33)	NR	ART: 17 (48.3) ART-naive: 16 (48.3)	ART: 41.9 ± 10.7 ART-naive: 43.6 ± 13.5	ART: 27.5 ± 6.4 ART-naive: 26.7 ± 5.2	385 ± 255 304 ± 190	There were no significant differences between the ART-exposed and ART-naive groups	Low
Moshomo et al., 2024 [38]	Botswana	2014 and 2015	Cross-sectional	ART (68) ART-naive (36)	9.8 ± 3.2 NR	ART: 33 (48.5) ART-naive: 15 (41.7)	ART: 38.7 ± 4.6 ART-naive: 37.8 ± 5.4	NR	540.2 ± 230.8 381.5 ± 236.6	There was no statistically significant difference in CIMT between the two groups	Low
Gupta et al., 2022 [20]	India	2020	Cross-sectional	ART (80) ART-naive (40)	NR	NR	ART: 37.09 ± 6.09 ART-naive: 33.85 ± 8.84	ART: 24.68 ± 3.18 ART-naive: 21.87 ± 2.84	357.38 ± 220.34 505.25 ± 268.97	CIMT was significantly higher in the ART groups than in the ART-naive group	Moderate
Castro et al., 2021 [39]	Colombia	2016 and 2017	Cross-sectional	ART (31) ART-naive (32)	6.5 ± 1.9 (78 ± 22.75) ^a^ 2.0 ± 0.93 (23.98 ± 11.1) ^a^	ART: 17 (55) ART-naive: 12 (63)	ART: 35.5 ± 9.8 ART-naive e: 30.2 ± 8.7	ART: 21.9 ± 0.835 ART-naive: 22.8 ± 0.83	705 ± 139.25 588.29 ± 274.26	There were no significant differences in CIMT between groups	Moderate
Vos et al., 2020 [21]	South Africa	2014 to 2017	Cross-sectional	HIV on ART (690) ART-naive (197)	70.58 ± 15.47 ^a^ (5.88 ± 1.29) 0.0 ± 1.17	ART: 285 (41.3) ART-naive: 73 (37.1)	ART: 41.2 ± 2.24 ART-naive: 35 ± 2.8	ART: 22.72 ± 1.21 ART-naive: 22.5 ± 1.27	490.98 ± 57.0 399 ± 46.33	There was a significant increase in CIMT in the ART group compared to the ART-naive group	Low
Yacovo et al., 2020 [48]	Spain	2012 and 2013	Prospective, longitudinal cohort	ART (31) ART-naive (30)	19.5 ± 9.26 ^a^ (1.63 ± 0.77) 23.1 ± 11.98 ^a^ (1.93 ± 0.99)	ART: 26 (83.9) ART-naive: 26 (83.9)	ART: 37.3 ± 7.99 ART-naive: 36.3 ± 9.06	ART: 24.5 ± 3.1 ART-naive: 24.6 ± 5.64	415 ± 216 734 ± 193	CIMT was lower in ART compared to ART-naive patients	Low
Sarfo et al., 2019 [49]	Ghana	NR	Cross-sectional	PLHIV on CART (250) CART-naive (201)	8.6 ± 4.4 1.3 ± 2.6	CART: 47 (18.8) CART-naive: 37 (18.4)	ART: 45.7 ± 8.6 CART-naive: 42.9 ± 8.8	CART: 27.1 ± 5.5 CART-naive: 24.5 ± 5.1	641.9 ± 331.5 315.9 ± 271.2	No significant differences in CIMT among CART, ART-naive patients	Low
Vos et al., 2019 [51]	South Africa	2016 to 2017	Cross-sectional	HIV on ART (287) ART-naive (104)	77.59 ± 27.73 ^a^ (6.47 ± 2.31) 0 ± 0.0	ART: 95 (32.6) ART-naive: 39 (37.5)	ART: 41.03 ± 8.03 ART-naive: 33.9 ± 8.4	ART: 25.62 ± 1.75 ART-naive: 23.6 ± 1.02	552.78 ± 112 281 ± 34.83	Significant increase in CIMT in ART compared to ART-naive patients	Low
Gupta et al., 2018 [60]	India	2013 and 2015	Cross-sectional	PLHIV on ART (124) ART-naive (44)	4.08 ± 2.58 1.63 ± 1.36	NR	ART: 32.96 ± 6.72 ART-naive: 33.25 ± 5.77	ART: 20.66 ± 2.53 ART-naive: 20.66 ± 2.15	330 ± 153 339 ± 213	CIMT was significantly higher in ART compared to ART-naive patients	Low
Kirichenko et al., 2018 [40]	Russia	NR	Cross-sectional	ART (25) ART-naive (11)	NR	NR	ART: 39 ± 7 ART-naive: 38 ± 6	NR	547 ± 261 460 ± 177	There was no significant difference in CIMT among the groups	Low
Vohra & Bharti, 2017 [57]	India	2015 to 2016	Case–control	ART (45) ART-naive (30)	5.6 ± 3.3 1.9 ± 1.6	ART: 23 (51.1) ART-naive: 19 (63.3)	ART: 39.2 ± 5.3 ART-naive: 35.5 ± 7.2	ART: 21.4 ± 3.3 ART-naive: 20.4 ± 4.2	443 ± 225 566 ± 207	CIMT was significantly higher in ART-treated than ART-naive individuals	Low
Gleason et al., 2015 [52]	Ethiopia	NR	Cross-sectional	HIV ART (230) ART-naive (51)	5.98 ± 0.61 1.6 ± 0.85	ART: 58 (25.2) ART-naive: 14 (27)	ART: 37.78 ± 1.99 ART-naive: 38 ± 3.25	ART: 22.02 ± 1.39 ART-naive: 22 ± 1.5	353.69 ± 63.45 395 ± 51	There were no significant differences in CIMT between the groups	Low
Goulenok et al., 2015 [42]	France	2008 to 2009	Cross-sectional	ART (30) ART-naive (29)	12.0 ± 4.9 6.3 ± 4.9	ART: 30 (100) ART-naive: 29 (100)	ART: 41.3 ± 5.9 ART-naive: 40.0 ± 7.6	ART: 23.8 ± 4.5 ART-naive: 23.8 ± 3.3	585 ± 136 454 ± 220	There were no significant differences in CIMT between the groups	Low
Fourie et al., 2015 [41]	South Africa	2005 to 2010	Observational cohort	PLHIV on ART (66) ART-naive (78)	5	ART: 17 (25.8) ART-naive: 27 (34.6)	ART: 48.5 ± 0.96 ART-naive: 48.0 ± 0.89	ART: 22.3 ± 0.76 ART-naive: 23.8 ± 0.69	398 ± 220 335 ± 192	CIMT did not show a significant difference between the groups	Low
Pirs et al., 2014 [54]	Slovenia	NR	Cross-sectional study	PLHIV on ART (60) ART-naive (26)	NR	ART: 60 (100) ART-naive: 26 (100)	ART: 39.3 ± 8.537 ART-naive: 37.9 ± 9.366	ART: 25.4 ± 3.436 ART-naive: 23.9 ± 2.119	269.9 ± 248.814 426.3 ± 199.414	CIMT increased significantly in ART compared to ART-naive patients	Low
Zhu et al., 2014 [43]	China	2013	Cross-sectional	PLHIV on HAART (50) HAART-naive (50)	14.68 ± 3.70 14.93 ± 3.82	HAART: 26 (52) HAART-naive: 25 (50)	ART: 49.43 ± 5.42 HAART-naive: 48.03 ± 7.81	ART: 21.57 ± 1.99 HAART-naive: 21.22 ± 1.97	413.07 ± 163.610 443.62 ± 213.647	There were no significant differences in CIMT between the therapy and untreated groups	Low
Desvarieux et al., 2013 [45]	USA	2008 and 2009	Cross-sectional	ART (50) ART-naive (50)	12.1 ± 5.2 5.8 ± 4.4	ART: 50 (100) ART-naive: 50 (100)	ART: 40.2 ± 6.2 ART-naive: 40.4 ± 7.3	ART: 23.5 ± 3.7 ART-naive: 23.8 ± 3.3	592 ± 187 429 ± 162	CIMT increased significantly in ART compared to ART-naive patients	Low
Suparna et al., 2013 [44]	India	NR	Cross-sectional	ART (26) ART-naive (16)	6.43 ± 4.11 2.2 ± 2.24	NR	ART: 38.6 ± 7.015 ART-naive: 34.13 ± 6.90	ART: 20.59 ± 3.2 ART-naive: 20.76 ± 4.2	437.62 ± 227.99 387.33 ± 317.9	There was no significant difference in CIMT between the groups	Moderate
Parrinello et al., 2012 [56]	United States of America	2004	Observational study	PLHIV on ART (374) ART-naive (227)	NR	ART: 0 (0) ART-naive: 0 (0)	ART: 42.1 ± 8.1 ART-naive: 40.3 ± 8.3	ART: 28.4 ± 6.9 ART-naive: 29.7 ± 8.3	508.04 ± 309.22 464.1 ± 280.3	CIMT increased significantly in ART compared to ART-naive patients	Low
Manfredi et al., 2011 [59]	Italy	2007	Cross-sectional	PLHIV on ART (16) ART-naive (11)	9.5 + 4.3 4.7 + 2.2	ART: 12 (75) ART-naive: 8 (72.7)	ART: 44 ± 21 ART-naive: 42 ± 18	ART: 24.4 + 10.8 ART-naive: 23.9 + 11.2	635 ± 299 530 ± 211	There was no significant difference in CIMT between the groups	Low
Brand et al., 2011 [53]	South Africa	NR	Cross-sectional	HIV HAART (12) HAART-naive (13)	NR	HAART: 10 (83.3) HAART-naive: 11 (84.6)	HAART: 46 ± 8 HAART-naive: 52 ± 11	HAART: 23.0 ± 5.0 HAART-naive: 24.5 ± 3.1	334 ± 95 316 ± 149	No significant decrease in CIMT in HAART compared to HAART-naive patients	Low
Cristofaro et al., 2011 [46]	Italy	2004 and 2008	Cross-sectional	HIV HAART (222) HAART-naive (64)	9 5	HAART: 134 (66.7) HAART-naive: 27 (42.2)	HAART: 43.8 ± 7.98 HAART-naive: 35.6 ± 7.26	NR	462 ± 342.83 293 ± 270.75	There was no significant difference in CIMT between the groups	Low
Calza et al., 2009 [55]	Italy	2007	Cross-sectional study	PLHIV on ART (45) ART-naive (21)	9.2 + 4.6 4.9 + 2.7	ART: 38 (84.4) ART-naive: 18 (85.7)	ART: 45 + 26 ART-naive: 43 ± 21	ART: 24.8 ± 11.2 ART-naive: 23.7 ± 11.5	656 ± 315 549 ± 254	Significantly higher CIMT in ART compared to ART-naive patients	Low
Van Vonderen et al., 2009 [47]	Netherlands	2004 and 2005	Case–control	ART (55) ART-naive (22)	NR	ART: 55 (100) ART-naive: 22 (100)	ART: 45.30 ± 4.3 ART-naive: 37.73 ± 1.63	ART: 23.316 ± 0.89 ART-naive: 23.75 ± 1.2	609.27 ± 106.9 431.25 ± 76.489	Significantly higher CIMT in ART compared to ART-naive patients	Low
Seminari et al., 2002 [58]	Italy	NR	Cross-sectional	ART (28) ART-naive (15)	NR	ART: 21 (75) ART-naive: 9 (60)	ART: 36 ± 7 ART-naive: 33 ± 5	ART: 22 ART-naive: 23	570 ± 258 534 ± 228	PI-treated patients had higher CIMT compared to both ART-naive patients	Low

ART: antiretroviral therapy, CART: combined antiretroviral therapy, CIMT: carotid intima-media thickness, HAART: highly active antiretroviral therapy, HIV: human immunodeficiency virus, NR: not reported, PI: protease inhibitor, PLHIV: people living with HIV, a: data reported as months.

## Data Availability

All data generated or analyzed during this study are included in this published article and its Supplementary Information files.

## Supplementary Materials

The following supporting information can be downloaded at https://www.mdpi.com/article/10.3390/v17070894/s1, Figure S1: Subgroup analysis for CIMT in ART compared to ART-naive patients. A. Subgroup analysis for CIMT in ART compared to ART-naive patients based on continent of publication. B. Subgroup analysis for CIMT in ART compared to ART-naive patients based on CD4 count for patients on ART. C. Subgroup analysis for CIMT in ART compared to ART-naive patients based on gender. D. Subgroup analysis for CIMT in ART compared to ART-naive patients based on study design. E. Subgroup analysis for CIMT in ART compared to ART-naive patients based on sample size. F. Subgroup analysis for CIMT in ART compared to ART-naive patients based on age. G. Subgroup analysis for CIMT in ART compared to ART-naive patients based on the risk of bias. H. Subgroup analysis for CIMT in ART compared to ART-naive patients based on WHO adoption of rapid ART initiation. I. Subgroup analysis based on the duration of HIV infection since diagnosis in PLHIV on ART. J. Subgroup analysis based on the duration of HIV infection since diagnosis in PLHIV on ART-naïve patients; Table S1: Search strategy used on databases; Table S2: Sensitivity analysis, showing the effect of the exclusion of one study on CIMT in PLHIV on ART compared to ART-naïve patients; Table S3: Quality assessment of cohort studies using the Newcastle–Ottawa scale; Table S4: Quality assessment of case–control studies using the Newcastle–Ottawa scale; Table S5: Quality assessment of cross-sectional studies using the Newcastle–Ottawa scale; Table S6: PRISMA checklist.

## Abbreviations

ART	Antiretroviral therapy
BMI	Body mass index
CIMT	Carotid-intima media thickness
CVD	Cardiovascular disease
HAART	Highly active antiretroviral therapy
HIV	Human immune viruses
Nef	Negative regulatory factors
PI	Protease inhibitors
PLHIV	People living with human immunodeficiency virus
ROS	Reactive oxygen species

## References

[1] So-Armah K., Benjamin L.A., Bloomfield G.S., Feinstein M.J., Hsue P., Njuguna B., Freiberg M.S. (2020). HIV and Cardiovascular Disease. Lancet HIV.

[2] HIV. https://www.who.int/data/gho/data/themes/hiv-aids.

[3] Ntinga X., Musiello F., Pita T., Mabaso N., Celum C., Szpiro A., van Rooyen H., Barnabas R., van Heerden A. (2024). People Living with HIV’s Perspectives of Acceptability of Fee for Home Delivery of ART: A Qualitative Study. BMC Health Serv. Res..

[4] Vemulapalli A.C., Elias A.A., Yerramsetti M.D., Olanisa O.O., Jain P., Khan Q.S., Butt S.R. (2023). The Impact of Contemporary Antiretroviral Drugs on Atherosclerosis and Its Complications in People Living With HIV: A Systematic Review. Cureus.

[5] Lacobellis G., Sharma A.M., Pellicelli A.M., Grisorio B., Barbarini G., Barbaro G. (2007). Epicardial Adipose Tissue Is Related to Carotid Intima-Media Thickness and Visceral Adiposity in HIV-Infected Patients with Highly Active Antiretroviral Therapy-Associated Metabolic Syndrome. Curr. HIV Res..

[6] Hsue Y., Lo J.C., Franklin A., Bolger A.F., Martin J.N., Deeks S.G., Waters D.D. (2004). Progression of Atherosclerosis as Assessed by Carotid Intima-Media Thickness in Patients with HIV Infection. Circulation.

[7] Delaney J.A.C., Scherzer R., Biggs M.L., Shliplak M.G., Polak J.F., Currier J.S., Kronmal R.A., Wanke C., Bacchetti P., O’leary D. (2010). Associations of Antiretroviral Drug Use and HIV-Specific Risk Factors with Carotid Intima–Media Thickness. AIDS.

[8] Strauss K.L.E., Phoswa W.N., Lebelo S.L., Modjadji P., Mokgalaboni K. (2024). Endothelial Dysfunction, a Predictor of Cardiovascular Disease in HIV Patients on Antiretroviral Therapy: A Systematic Review and Meta-Analysis. Thromb. Res..

[9] Zanella I., Biasiotto G., Castelli F., Calza S., Carriero C., Degli Antoni M., Focà E., Quiros-Roldan E. (2021). Descriptive Modification of Inflammatory Markers in HIV Patients after CART Initiation According to Gender, Smoking Habit, CMV Infection, BMI and Serum Lipids. Cytokine.

[10] The DAD Study Group (2007). Class of Antiretroviral Drugs and the Risk of Myocardial Infarction. N. Engl. J. Med..

[11] Jaschinski N., Greenberg L., Neesgaard B., Miró J.M., Grabmeier-Pfistershammer K., Wandeler G., Smith C., De Wit S., Wit F., Pelchen-Matthews A. (2023). Recent Abacavir Use and Incident Cardiovascular Disease in Contemporary-Treated People with HIV. AIDS.

[12] Adedokun T.A., Kwaghe V.G., Adedokun O., Badru T., Odili A.N., Alfa J., Kolade-Yunusa H.O., Ojji D.B. (2023). Prevalence and Risk Factors for Subclinical Atherosclerosis amongst Adults Living with HIV in University of Abuja Teaching Hospital, Gwagwalada. Front. Reprod. Health.

[13] Mclaughlin M.M., Ma Y., Scherzer R., Rahalkar S., Martin J.N., Mills C., Milush J., Deeks S.G., Hsue P.Y. (2020). Association of Viral Persistence and Atherosclerosis in Adults with Treated HIV Infection. JAMA Netw. Open.

[14] Hyle E.P., Mayosi B.M., Middelkoop K., Mosepele M., Martey E.B., Walensky R.P., Bekker L.G., Triant V.A. (2017). The Association between HIV and Atherosclerotic Cardiovascular Disease in Sub-Saharan Africa: A Systematic Review. BMC Public Health.

[15] Calza L., Manfredi R., Colangeli V., Trapani F.F., Salvadori C., Magistrelli E., Danese I., Verucchi G., Serra C., Viale P. (2013). Two-Year Treatment with Rosuvastatin Reduces Carotid Intima-Media Thickness in HIV Type 1-Infected Patients Receiving Highly Active Antiretroviral Therapy with Asymptomatic Atherosclerosis and Moderate Cardiovascular Risk. AIDS Res. Hum. Retroviruses.

[16] Nou E., Lo J., Grinspoon S.K. (2016). Inflammation, Immune Activation, and Cardiovascular Disease in HIV. AIDS.

[17] Vos A.G., Dodd C.N., Delemarre E.M., Nierkens S., Serenata C., Grobbee D.E., Klipstein-Grobusch K., Venter W.D.F. (2021). Patterns of Immune Activation in HIV and Non HIV Subjects and Its Relation to Cardiovascular Disease Risk. Front. Immunol..

[18] Lorenz M.W., Stephan C., Harmjanz A., Staszewski S., Buehler A., Bickel M., von Kegler S., Ruhkamp D., Steinmetz H., Sitzer M. (2008). Both Long-Term HIV Infection and Highly Active Antiretroviral Therapy Are Independent Risk Factors for Early Carotid Atherosclerosis. Atherosclerosis.

[19] Liu X., Sun Y., Zhan Y., Jiang Y. (2021). Prevalence and Risk of Subclinical Carotid Atherosclerosis in the Global Population with HIV: A Systematic Review and Meta-Analysis. Int. J. STD AIDS.

[20] Gupta P.K., Tyagi S., Sheoran A., Jain P., Koner S.K., Sharma L.K., Singh S.K., Khura J. (2022). Effect of Antiretroviral Therapy on Cardiac Risk Markers in People Living with HIV/AIDS. Indian J. Sex. Transm. Dis. AIDS.

[21] Vos A.G., Barth R.E., Klipstein-Grobusch K., Tempelman H.A., Devillé W.L.J., Dodd C., Coutinho R.A., Grobbee D.E. (2020). Cardiovascular Disease Burden in Rural Africa: Does Hiv and Antiretroviral Treatment Play a Role?. J. Am. Heart Assoc..

[22] Majonga E.D., Chiesa S.T., McHugh G., Mujuru H., Nathoo K., Odland J.O., Kaski J.P., Ferrand R.A. (2020). Carotid Intima Media Thickness in Older Children and Adolescents with HIV Taking Antiretroviral Therapy. Medicine.

[23] Nalado A.M., Waziri B., Ismail A., Umar N., Ibrahim Z.U., Obiagwu P., Musa B.M., Sani M.U., Abdu A., Dankishiya F.S. (2023). Prevalence and Determinants of Endothelial Dysfunction among Adults Living with HIV in Northwest Nigeria. Glob. Heart.

[24] Sadiq E., Woodiwiss A., Tade G., Norton G., Modi G. (2024). Lack of Impact of HIV Status on Carotid Intima Media Thickness in a Cohort of Stroke Patients in South Africa. J. Neurol. Sci..

[25] Majonga E.D., Ferrand R.A., Deanfield J.E., Chiesa S.T. (2022). The Effect of Perinatal HIV and Antiretroviral Therapy on Vascular Structure and Function in Young People: A Systematic Review and Meta-Analysis. Atherosclerosis.

[26] Msoka T.F., Van Guilder G.P., van Furth M., Smulders Y., Meek S.J., Bartlett J.A., Vissoci J.R.N., van Agtmael M.A. (2019). The Effect of HIV Infection, Antiretroviral Therapy on Carotid Intima-Media Thickness: A Systematic Review and Meta-Analysis. Life Sci..

[27] Page M.J., McKenzie J.E., Bossuyt P.M., Boutron I., Hoffmann T.C., Mulrow C.D., Shamseer L., Tetzlaff J.M., Akl E.A., Brennan S.E. (2021). The PRISMA 2020 Statement: An Updated Guideline for Reporting Systematic Reviews. BMJ.

[28] Why the HIV Epidemic Is Not Over. https://www.who.int/news-room/spotlight/why-the-hiv-epidemic-is-not-over.

[29] McHugh M.L. (2012). Interrater Reliability: The Kappa Statistic. Biochem. Med..

[30] Wells G., Shea B., O’Connell D., Peterson J., Welch V., Losos M., Tugwell P. (2012). The Newcastle-Ottawa Scale (NOS) for Assessing the Quality If Nonrandomized Studies in Meta-Analyses. http://www.ohri.ca/programs/clinical_epidemiology/oxford.asp.

[31] Hozo S.P., Djulbegovic B., Hozo I. (2005). Estimating the Mean and Variance from the Median, Range, and the Size of a Sample. BMC Med. Res. Methodol..

[32] Higgins J.P.T., Thompson S.G. (2002). Quantifying Heterogeneity in a Meta-Analysis. Stat. Med..

[33] Doleman B., Freeman S.C., Lund J.N., Williams J.P., Sutton A.J. (2020). Funnel Plots May Show Asymmetry in the Absence of Publication Bias with Continuous Outcomes Dependent on Baseline Risk: Presentation of a New Publication Bias Test. Res. Synth. Methods.

[34] Egger M., Smith G.D., Schneider M., Minder C. (1997). Bias in Meta-Analysis Detected by a Simple, Graphical Test. BMJ.

[35] Sedgwick P. (2013). Meta-Analyses: Heterogeneity and Subgroup Analysis. BMJ.

[36] Mathur M.B., VanderWeele T.J. (2020). Sensitivity Analysis for Publication Bias in Meta-Analyses. J. R. Stat. Soc. Ser. C Appl. Stat..

[37] Fekete J.T., Győrffy B. (2025). MetaAnalysisOnline.Com: Web-Based Tool for the Rapid Meta-Analysis of Clinical and Epidemiological Studies. J. Med. Internet Res..

[38] Moshomo T., Molefe-Baikai O.J., Bennett K., Gaolathe T., Moyo S., Gaseitsewe S., Mohammed T., Lockman S., Mosepele M. (2024). Cytomegalovirus Immunoglobulin G Levels and Subclinical Arterial Disease among People Living with HIV in Botswana: A Cross-Sectional Study. Biomedicines.

[39] Castro G., León K., Marín-Palma D., Oyuela S.M., Cataño-Bedoyam J.U., Duque-Botero J., Giraldo-Méndez D.P., Taborda N.A., Hernandez J.C., Rugeles M.T. (2021). Evaluation of Differences in Metabolic and Immunologic Markers and Cardiovascular Risk in Hiv-1 Patients. Rev. Cienc. Salud.

[40] Kirichenko T.V., Myasoedova V.A., Shimonova T.E., Melnichenko A.A., Sviridov D., Sobenin I.A., Mazus A.I., Orekhov A.N., Bukrinsky M.I. (2018). Atherosclerosis in Subjects Newly Diagnosed with Human Immunodeficiency Virus Infection. Biosci. Rep..

[41] Fourie C.M.T., Schutte A.E., Smith W., Kruger A., van Rooyen J.M. (2015). Endothelial Activation and Cardiometabolic Profiles of Treated and Never-Treated HIV Infected Africans. Atherosclerosis.

[42] Goulenok T., Boyd A., Larsen M., Fastenackels S., Boccara F., Meynard J.L., Hadour N., Samri A., Desvarieux M., Autrana B. (2015). Increased Carotid Intima-Media Thickness Is Not Associated with T-Cell Activation nor with Cytomegalovirus in HIV-Infected Neversmoker Patients. AIDS.

[43] Zhu H., Yuan J., Wang Y., Gao F., Wang X., Wei C., Chen J., Fan X., Zhang M. (2014). Long-Term Use of First-Line Highly Active Antiretroviral Therapy Is Not Associated with Carotid Artery Stiffness in Human Immunodeficiency Virus-Positive Patients. Braz. J. Infect. Dis..

[44] Suparna P.N., Achappa B., Unnikrishnan B., Madi D., Chowta M.N., Ramapuram J.T., Rao S., Mahalingam S. (2013). The Evaluation of Carotid Atherosclerosis in Patients with the HIV-1 Infection: The Role of the Antiretroviral Therapy. J. Clin. Diagn. Res..

[45] Desvarieux M., Boccara F., Meynard J.L., Bastard J.P., Mallat Z., Charbit B., Demmer R.T., Haddour N., Fellahi S., Tedgui A. (2013). Infection Duration and Inflammatory Imbalance Are Associated with Atherosclerotic Risk in HIV-Infected Never-Smokers Independent of Antiretroviral Therapy. AIDS.

[46] Cristofaro M., Cicalini S., Busi Rizzi E., Schininà V., Petrosillo N., Bibbolino C. (2011). Valutazione Ecografica Delle Lesioni Vascolari Carotidee Nei Pazienti HIV Positivi. Radiol. Medica.

[47] Van Vonderen M.G.A., Smulders Y.M., Stehouwer C.D.A., Danner S.A., Gundy C.M., Vos F., Reiss P., Van Agtmael M.A. (2009). Carotid Intima-Media Thickness and Arterial Stiffness in HIV-Infected Patients: The Role of HIV, Antiretroviral Therapy, and Lipodystrophy. J. Acquir. Immune Defic. Syndr..

[48] Di Yacovo S., Saumoy M., Sánchez-Quesada J.L., Navarro A., Sviridov D., Javaloyas M., Vila R., Vernet A., Low H., Peñafiel J. (2020). Lipids, Biomarkers, and Subclinical Atherosclerosis in Treatment-Naive HIV Patients Starting or Not Starting Antiretroviral Therapy: Comparison with a Healthy Control Group in a 2-Year Prospective Study. PLoS ONE.

[49] Sarfo F.S., Nichols M., Agyei B., Singh A., Ennin E., Nyantakyi A.D., Asibey S.O., Tagge R., Gebregziabher M., Jenkins C. (2019). Burden of Subclinical Carotid Atherosclerosis and Vascular Risk Factors among People Living with HIV in Ghana. J. Neurol. Sci..

[50] Chikwati R.P., Jaff N.G., Mahyoodeen N.G., Micklesfield L.K., Ramsay M., Gómez-Olivé F.X., Mohamed S.F., Choma S.S.R., George J.A., Crowther N.J. (2024). The Association of Menopause with Cardiometabolic Disease Risk Factors in Women Living with and without HIV in Sub-Saharan Africa: Results from the AWI-Gen 1 Study. Maturitas.

[51] Vos A.G., Hoeve K., Barth R.E., Peper J., Moorhouse M., Crowther N.J., Venter W.D.F., Grobbee D.E., Bots M.L., Klipstein-Grobusch K. (2019). Cardiovascular Disease Risk in an Urban African Population: A Cross-Sectional Analysis on the Role of HIV and Antiretroviral Treatment. Retrovirology.

[52] Gleason R.L., Caulk A.W., Seifu D., Parker I., Vidakovic B., Getenet H., Assefa G., Amogne W. (2015). Current Efavirenz (EFV) or Ritonavir-Boosted Lopinavir (LPV/r) Use Correlates with Elevate Markers of Atherosclerosis in HIV-Infected Subjects in Addis Ababa, Ethiopia. PLoS ONE.

[53] Brand M., Woodiwiss A.J., Michel F., Booysen H.L., Majane O.H.I., Maseko M.J., Veller M.G., Norton G.R. (2012). Carotid Intima-Media Thickness in African Patients with Critical Lower Limb Ischemia Infected with the Human Immunodeficiency Virus. J. AIDS Clin. Res..

[54] Pirs M., Eržen B., Šabović M., Karner P., Vidmar L., Poljak M., Jug B., Mikac M., Tomažič J. (2014). Early Atherosclerosis in HIV-Infected Patients below the Age of 55 Years: Slovenian National Study. Wien. Klin. Wochenschr..

[55] Calza L., Verucchi G., Pocaterra D., Pavoni M., Alfieri A., Cicognani A., Manfredi R., Serra C., Chiodo F. (2009). Cardiovascular Risk Factors and Ultrasound Evaluation of Carotid Atherosclerosis in Patients with HIV-1 Infection. Int. J. STD AIDS.

[56] Parrinello C.M., Sinclair E., Landay A.L., Lurain N., Sharrett A.R., Gange S.J., Xue X., Hunt P.W., Deeks S.G., Hodis H.N. (2012). Cytomegalovirus Immunoglobulin G Antibody Is Associated with Subclinical Carotid Artery Disease among HIV-Infected Women. J. Infect. Dis..

[57] Vohra S., Bharti V., Sharma A., Jaret P.K., Marwaha R., Balraj, Sachdeva A. (2017). Comparison of CIMT among HIV Seropositive and HIV Seronegative Subjects: A Case-Control Study. Int. J. Curr. Adv. Res..

[58] Seminari E., Pan A., Voltini G., Carnevale G., Maserati R., Minoli L., Meneghetti G., Tinelli C., Testa S. (2002). Assessment of Atherosclerosis Using Carotid Ultrasonography in a Cohort of HIV-Positive Patients Treated with Protease Inhibitors. Atherosclerosis.

[59] Manfredi R. (2011). HIV Disease, Antiretroviral Therapy Safety and the Cardiovascular System. Clinical-Instrumental Assessment of Antiretroviral-Naïve Versus Subjects Already Treated with Antiretroviral Agents. Open Drug Saf. J..

[60] Gupta P.K., Gupta M., Lal A.K., Taneja A., Taneja R.S., Rewari B.B. (2018). Markers of Subclinical Atherosclerotic Disease in HIV-Infected Individuals. J. Virus Erad..

[61] Joseph J., Boban S.M. (2025). Study of Cardiovascular Risks and Carotid Intimal Thickness (CIMT) And The Effect Of Antiretroviral Therapy In The Cardiovascular Profile Among HIV Patients. Int. J. Sci. Res..

[62] Ozdemir S., Ozdemir E., Birlik B., Demirdal T. (2021). The Value of Carotid Intima-Media Thickness in the Detection of Atherosclerosis in HIV^+^ Patients Subclinical Atherosclerosis in HIV^+^. J. Coll. Physicians Surg. Pak..

[63] Li J.Z., Segal F.P., Bosch R.J., Lalama C.M., Roberts-Toler C., Delagreverie H., Getz R., Garcia-Broncano P., Kinslow J., Tressler R. (2020). Antiretroviral Therapy Reduces T-Cell Activation and Immune Exhaustion Markers in Human Immunodeficiency Virus Controllers. Clin. Infect. Dis..

[64] Strauss K.-L.E., Phoswa W.N., Mokgalaboni K. (2024). The Impact of Antiretroviral Therapy on Liver Function Among 2 HIV Infected Pregnant Women with and Without Pre-Eclampsia. Viruses.

[65] Obare L.M., Temu T., Mallal S.A., Wanjalla C.N. (2024). Inflammation in HIV and Its Impact on Atherosclerotic Cardiovascular Disease. Circ Res.

[66] Kearns A., Gordon J., Burdo T.H., Qin X. (2017). HIV-1–Associated Atherosclerosis: Unraveling the Missing Link. J. Am. Coll. Cardiol..

[67] Qu B., Qu T. (2015). Causes of Changes in Carotid Intima-Media Thickness: A Literature Review. Cardiovasc. Ultrasound.

[68] Alfaddagh A., Martin S.S., Leucker T.M., Michos E.D., Blaha M.J., Lowenstein C.J., Jones S.R., Toth P.P. (2020). Inflammation and Cardiovascular Disease: From Mechanisms to Therapeutics. Am. J. Prev. Cardiol..

[69] Harshithkumar R., Shah P., Jadaun P., Mukherjee A. (2024). ROS Chronicles in HIV Infection: Genesis of Oxidative Stress, Associated Pathologies, and Therapeutic Strategies. Curr. Issues Mol. Biol..

[70] Nzuza S., Zondi S., Hurchund R., Owira P.M. (2017). Highly Active Antiretroviral Therapy-Associated Metabolic Syndrome and Lipodystrophy: Pathophysiology and Current Therapeutic Interventions. J. Endocrinol. Metab..

[71] Dirajlal-Fargo S., Funderburg N. (2022). HIV and Cardiovascular Disease: The Role of Inflammation. Curr. Opin. HIV AIDS.

[72] Hunt P.W. (2017). Very Early ART and Persistent Inflammation in Treated HIV. Clin. Infect. Dis..

[73] Di Pino A., Defronzo R.A. (2019). Insulin Resistance and Atherosclerosis: Implications for Insulin-Sensitizing Agents. Endocr. Rev..

[74] The RESPOND Study Group (2021). Incidence of Dyslipidemia in People with HIV Who Are Treated with Integrase Inhibitors versus Other Antiretroviral Agents. AIDS.

[75] Dressman J., Kincer J., Matveev S.V., Guo L., Greenberg R.N., Guerin T., Meade D., Li X.-A., Zhu W., Uittenbogaard A. (2003). HIV Protease Inhibitors Promote Atherosclerotic Lesion Formation Independent of Dyslipidemia by Increasing CD36-Dependent Cholesteryl Ester Accumulation in Macrophages. J. Clin. Investig..

[76] Velichkovska M., Surnar B., Nair M., Dhar S., Toborek M. (2019). Targeted Mitochondrial COQ 10 Delivery Attenuates Antiretroviral-Drug-Induced Senescence of Neural Progenitor Cells. Mol. Pharm..

[77] Lombardi F., Belmonti S., Sanfilippo A., Borghetti A., Iannone V., Salvo P.F., Fabbiani M., Visconti E., Giambenedetto S. (2024). Di Factors Associated with Oxidative Stress in Virologically Suppressed People Living with HIV on Long-Term Antiretroviral Therapy. AIDS Res. Ther..

[78] Ivanov A.V., Valuev-Elliston V.T., Ivanova O.N., Kochetkov S.N., Starodubova E.S., Bartosch B., Isaguliants M.G. (2016). Oxidative Stress during HIV Infection: Mechanisms and Consequences. Oxid. Med. Cell Longev..

[79] Alikhani A., Morin H., Matte S., Alikhani P., Tremblay C., Durand M. (2019). Association between Lipodystrophy and Length of Exposure to ARTs in Adult HIV-1 Infected Patients in Montreal. BMC Infect. Dis..

[80] Buthelezi L.M., Munsamy A.J., Mashige K.P. (2024). Inflammatory Mechanisms Contributing to Retinal Alterations in HIV Infection and Long-Term ART. S. Afr. J. HIV Med..

[81] Vos A.G., Venter W.D.F. (2021). Cardiovascular Toxicity of Contemporary Antiretroviral Therapy. Curr. Opin. HIV AIDS.

[82] Babu H., Ambikan A.T., Gabriel E.E., Akusjärvi S.S., Palaniappan A.N., Sundaraj V., Mupanni N.R., Sperk M., Cheedarla N., Sridhar R. (2019). Systemic Inflammation and the Increased Risk of Inflamm-Aging and Age-Associated Diseases in People Living with HIV on Long Term Suppressive Antiretroviral Therapy. Front. Immunol..

[83] Wu T.W., Hung C.L., Liu C.C., Wu Y.J., Wang L.Y., Yeh H.I. (2017). Associations of Cardiovascular Risk Factors with Carotid Intima-Media Thickness in Middle-Age Adults and Elders. J. Atheroscler. Thromb..

[84] Vickhoff B. (2023). Why Art? The Role of Arts in Arts and Health. Front. Psychol..

[85] Millard E., Medlicott E., Cardona J., Priebe S., Carr C. (2021). Preferences for Group Arts Therapies: A Cross-Sectional Survey of Mental Health Patients and the General Population. BMJ Open.

[86] González-Cordón A., Assoumou L., Camafort M., Domenech M., Guaraldi G., Domingo P., Rusconi S., Raffi F., Katlama C., Masia M. (2020). Switching from Boosted PIs to Dolutegravir in HIV-Infected Patients with High Cardiovascular Risk: 48 Week Effects on Subclinical Cardiovascular Disease. J. Antimicrob. Chemother..

