# Supplementary material for: The Association Between HIV Infection and Carotid Intima-Media Thickness in the Era of Antiretroviral Therapy: A Meta-Analysis
Source: Viruses. 2025 Jun 25;17(7):894. doi: 10.3390/v17070894 (PMC12298079; doi:10.3390/v17070894)
Supplement: Supplementary file 1 [file viruses-17-00894-s001.zip › viruses-3716372-supplementary s1.pdf]

The Association Between HIV Infection and Carotid Intima-Media Thickness in the Era of Antiretroviral Therapy: A Meta-Analysis

Table S1: Search strategy used on databases

Database	Search	Number
PubMed	(((Antiretroviral therapy) OR (combined antiretroviral therapy)) AND (HIV)) AND (carotid intima media thickness)	271
Scopus	(TITLE-ABS-KEY (carotid AND intima AND media AND thickness) AND TITLE-ABS-KEY (hiv) AND TITLE-ABS-KEY (carotid AND intima AND media AND thickness)) AND (LIMIT-TO (SRCTYPE , "j")) AND (LIMIT-TO (DOCTYPE , "ar")) AND (LIMIT-TO (LANGUAGE , "English"))	390
EBSCOhost	((Title Combined:(Antiretroviral therapy)) OR (TitleCombined:(Highly active antiretroviral therapy))) AND (HIV) AND (Carotid intima media thickness)	59

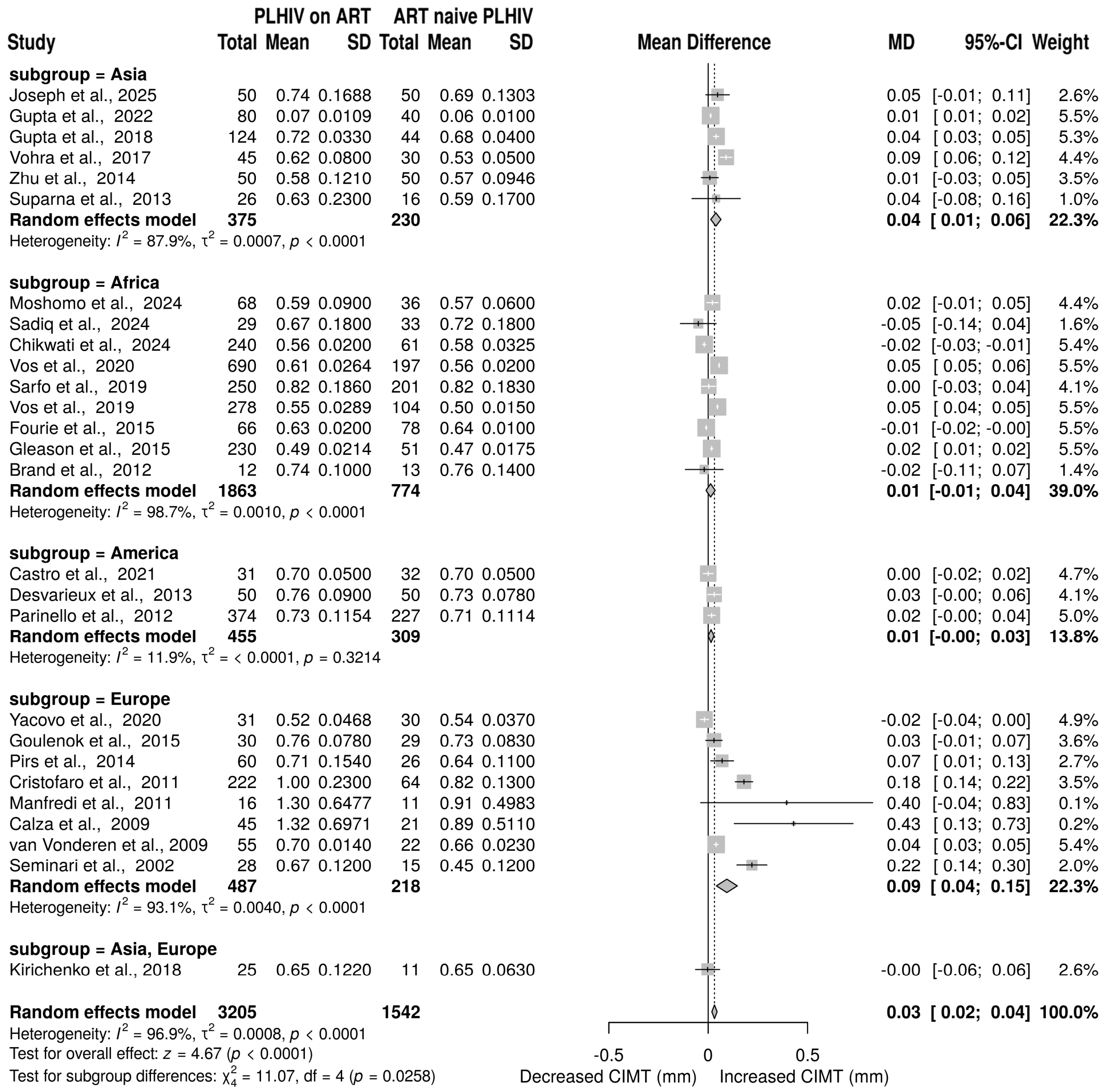
PLHIV on ART ART naive PLHIV
Study Total Mean SD Total Mean SD
Mean Difference
MD 95%-CI Weight
subgroup = Asia
Joseph et al., 2025 50 0.74 0.1688 50 0.69 0.1303 0.05 [-0.01; 0.11] 2.6%
Gupta et al., 2022 80 0.07 0.0109 40 0.06 0.0100 0.01 [0.01; 0.02] 5.5%
Gupta et al., 2018 124 0.72 0.0330 44 0.68 0.0400 0.04 [0.03; 0.05] 5.3%
Vohra et al., 2017 45 0.62 0.0800 30 0.53 0.0500 0.09 [0.06; 0.12] 4.4%
Zhu et al., 2014 50 0.58 0.1210 50 0.57 0.0946 0.01 [-0.03; 0.05] 3.5%
Suparna et al., 2013 26 0.63 0.2300 16 0.59 0.1700 0.04 [-0.08; 0.16] 1.0%
Random effects model 375 230 0.04 [0.01; 0.06] 22.3%
Heterogeneity: I^2 = 87.9%, τ^2 = 0.0007, p < 0.0001
subgroup = Africa
Moshomo et al., 2024 68 0.59 0.0900 36 0.57 0.0600 0.02 [-0.01; 0.05] 4.4%
Sadiq et al., 2024 29 0.67 0.1800 33 0.72 0.1800 -0.05 [-0.14; 0.04] 1.6%
Chikwati et al., 2024 240 0.56 0.0200 61 0.58 0.0325 -0.02 [-0.03; -0.01] 5.4%
Vos et al., 2020 690 0.61 0.0264 197 0.56 0.0200 0.05 [0.05; 0.06] 5.5%
Sarfo et al., 2019 250 0.82 0.1860 201 0.82 0.1830 0.00 [-0.03; 0.04] 4.1%
Vos et al., 2019 278 0.55 0.0289 104 0.50 0.0150 0.05 [0.04; 0.05] 5.5%
Fourie et al., 2015 66 0.63 0.0200 78 0.64 0.0100 -0.01 [-0.02; -0.00] 5.5%
Gleason et al., 2015 230 0.49 0.0214 51 0.47 0.0175 0.02 [0.01; 0.02] 5.5%
Brand et al., 2012 12 0.74 0.1000 13 0.76 0.1400 -0.02 [-0.11; 0.07] 1.4%
Random effects model 1863 774 0.01 [-0.01; 0.04] 39.0%
Heterogeneity: I^2 = 98.7%, τ^2 = 0.0010, p < 0.0001
subgroup = America
Castro et al., 2021 31 0.70 0.0500 32 0.70 0.0500 0.00 [-0.02; 0.02] 4.7%
Desvarieux et al., 2013 50 0.76 0.0900 50 0.73 0.0780 0.03 [-0.00; 0.06] 4.1%
Parinello et al., 2012 374 0.73 0.1154 227 0.71 0.1114 0.02 [-0.00; 0.04] 5.0%
Random effects model 455 309 0.01 [-0.00; 0.03] 13.8%
Heterogeneity: I^2 = 11.9%, τ^2 = < 0.0001, p = 0.3214
subgroup = Europe
Yacovo et al., 2020 31 0.52 0.0468 30 0.54 0.0370 -0.02 [-0.04; 0.00] 4.9%
Goulenok et al., 2015 30 0.76 0.0780 29 0.73 0.0830 0.03 [-0.01; 0.07] 3.6%
Pirs et al., 2014 60 0.71 0.1540 26 0.64 0.1100 0.07 [0.01; 0.13] 2.7%
Cristofaro et al., 2011 222 1.00 0.2300 64 0.82 0.1300 0.18 [0.14; 0.22] 3.5%
Manfredi et al., 2011 16 1.30 0.6477 11 0.91 0.4983 0.40 [-0.04; 0.83] 0.1%
Calza et al., 2009 45 1.32 0.6971 21 0.89 0.5110 0.43 [0.13; 0.73] 0.2%
van Vonderen et al., 2009 55 0.70 0.0140 22 0.66 0.0230 0.04 [0.03; 0.05] 5.4%
Seminari et al., 2002 28 0.67 0.1200 15 0.45 0.1200 0.22 [0.14; 0.30] 2.0%
Random effects model 487 218 0.09 [0.04; 0.15] 22.3%
Heterogeneity: I^2 = 93.1%, τ^2 = 0.0040, p < 0.0001
subgroup = Asia, Europe
Kirichenko et al., 2018 25 0.65 0.1220 11 0.65 0.0630 -0.00 [-0.06; 0.06] 2.6%
Random effects model 3205 1542 0.03 [0.02; 0.04] 100.0%
Heterogeneity: I^2 = 96.9%, τ^2 = 0.0008, p < 0.0001
Test for overall effect: z = 4.67 (p < 0.0001)
Test for subgroup differences: χ^2_4 = 11.07, df = 4 (p = 0.0258)
-0.5 0 0.5
Decreased CIMT (mm) Increased CIMT (mm)

A

Study	PLHIV on ART Total	PLHIV on ART Mean	PLHIV on ART SD	ART naive PLHIV Total	ART naive PLHIV Mean	ART naive PLHIV SD	MD	95%-CI	Weight
**subgroup = NR**									
Joseph et al., 2025	50	0.74	0.1688	50	0.69	0.1303	0.05	[-0.01; 0.11]	2.6%
Chikwati et al., 2024	240	0.56	0.0200	61	0.58	0.0325	-0.02	[-0.03; -0.01]	5.4%
**Random effects model**	**290**			**111**			**0.01**	**[-0.06; 0.07]**	**8.1%**
Heterogeneity: I^2 = 79.4%, τ^2 = 0.0018, p = 0.0274									
**subgroup = CD4 greater than 500**									
Moshomo et al., 2024	68	0.59	0.0900	36	0.57	0.0600	0.02	[-0.01; 0.05]	4.4%
Castro et al., 2021	31	0.70	0.0500	32	0.70	0.0500	0.00	[-0.02; 0.02]	4.7%
Sarfo et al., 2019	250	0.82	0.1860	201	0.82	0.1830	0.00	[-0.03; 0.04]	4.1%
Vos et al., 2019	278	0.55	0.0289	104	0.50	0.0150	0.05	[0.04; 0.05]	5.5%
Kirichenko et al., 2018	25	0.65	0.1220	11	0.65	0.0630	-0.00	[-0.06; 0.06]	2.6%
Goulenok et al., 2015	30	0.76	0.0780	29	0.73	0.0830	0.03	[-0.01; 0.07]	3.6%
Desvarieux et al., 2013	50	0.76	0.0900	50	0.73	0.0780	0.03	[-0.00; 0.06]	4.1%
Parinello et al., 2012	374	0.73	0.1154	227	0.71	0.1114	0.02	[-0.00; 0.04]	5.0%
Manfredi et al., 2011	16	1.30	0.6477	11	0.91	0.4983	0.40	[-0.04; 0.83]	0.1%
Calza et al., 2009	45	1.32	0.6971	21	0.89	0.5110	0.43	[0.13; 0.73]	0.2%
van Vonderen et al., 2009	55	0.70	0.0140	22	0.66	0.0230	0.04	[0.03; 0.05]	5.4%
Seminari et al., 2002	28	0.67	0.1200	15	0.45	0.1200	0.22	[0.14; 0.30]	2.0%
**Random effects model**	**1250**			**759**			**0.03**	**[0.02; 0.05]**	**41.6%**
Heterogeneity: I^2 = 82.4%, τ^2 = 0.0005, p < 0.0001									
**subgroup = CD4 less than 500**									
Sadiq et al., 2024	29	0.67	0.1800	33	0.72	0.1800	-0.05	[-0.14; 0.04]	1.6%
Gupta et al., 2022	80	0.07	0.0109	40	0.06	0.0100	0.01	[0.01; 0.02]	5.5%
Yacovo et al., 2020	31	0.52	0.0468	30	0.54	0.0370	-0.02	[-0.04; 0.00]	4.9%
Vos et al., 2020	690	0.61	0.0264	197	0.56	0.0200	0.05	[0.05; 0.06]	5.5%
Gupta et al., 2018	124	0.72	0.0330	44	0.68	0.0400	0.04	[0.03; 0.05]	5.3%
Vohra et al., 2017	45	0.62	0.0800	30	0.53	0.0500	0.09	[0.06; 0.12]	4.4%
Fourie et al., 2015	66	0.63	0.0200	78	0.64	0.0100	-0.01	[-0.02; -0.00]	5.5%
Gleason et al., 2015	230	0.49	0.0214	51	0.47	0.0175	0.02	[0.01; 0.02]	5.5%
Pirs et al., 2014	60	0.71	0.1540	26	0.64	0.1100	0.07	[0.01; 0.13]	2.7%
Zhu et al., 2014	50	0.58	0.1210	50	0.57	0.0946	0.01	[-0.03; 0.05]	3.5%
Suparna et al., 2013	26	0.63	0.2300	16	0.59	0.1700	0.04	[-0.08; 0.16]	1.0%
Brand et al., 2012	12	0.74	0.1000	13	0.76	0.1400	-0.02	[-0.11; 0.07]	1.4%
Cristofaro et al., 2011	222	1.00	0.2300	64	0.82	0.1300	0.18	[0.14; 0.22]	3.5%
**Random effects model**	**1665**			**672**			**0.03**	**[0.01; 0.05]**	**50.3%**
Heterogeneity: I^2 = 98%, τ^2 = 0.0009, p < 0.0001									
**Random effects model**	**3205**			**1542**			**0.03**	**[0.02; 0.04]**	**100.0%**

Heterogeneity: I^2 = 96.9%, τ^2 = 0.0008, p < 0.0001
Test for overall effect: z = 4.67 (p < 0.0001)
Test for subgroup differences: χ^2_2 = 0.62, df = 2 (p = 0.7347)

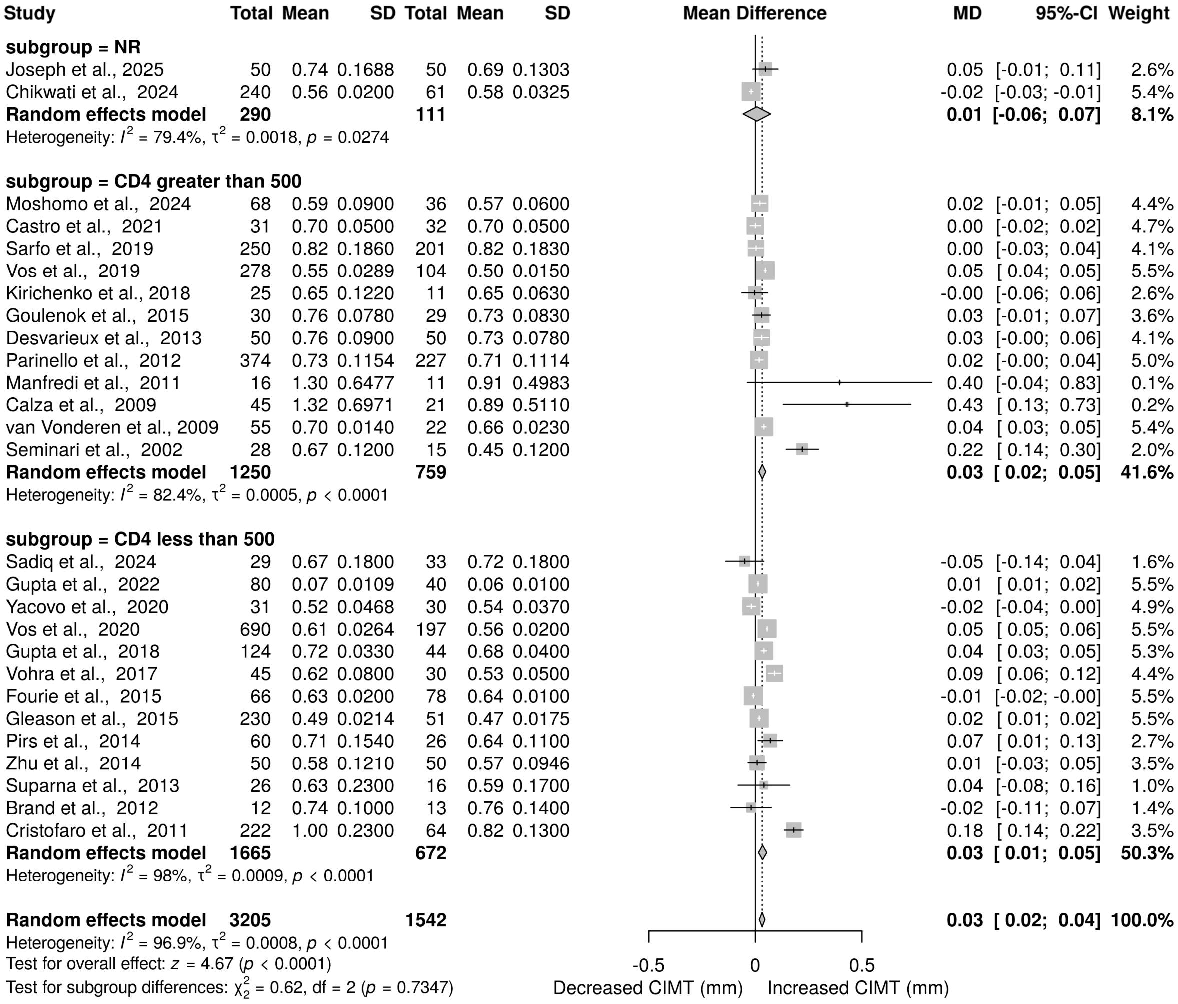

B

Study	PLHIV on ART Total	PLHIV on ART Mean	PLHIV on ART SD	ART naive PLHIV Total	ART naive PLHIV Mean	ART naive PLHIV SD	MD	95%-CI	Weight
**subgroup = NR**									
Joseph et al., 2025	50	0.74	0.1688	50	0.69	0.1303	0.05	[-0.01; 0.11]	2.6%
**subgroup = Both**									
Moshomo et al., 2024	68	0.59	0.0900	36	0.57	0.0600	0.02	[-0.01; 0.05]	4.4%
Sadiq et al., 2024	29	0.67	0.1800	33	0.72	0.1800	-0.05	[-0.14; 0.04]	1.6%
Gupta et al., 2022	80	0.07	0.0109	40	0.06	0.0100	0.01	[0.01; 0.02]	5.5%
Castro et al., 2021	31	0.70	0.0500	32	0.70	0.0500	0.00	[-0.02; 0.02]	4.7%
Yacovo et al., 2020	31	0.52	0.0468	30	0.54	0.0370	-0.02	[-0.04; 0.00]	4.9%
Vos et al., 2020	690	0.61	0.0264	197	0.56	0.0200	0.05	[0.05; 0.06]	5.5%
Sarfo et al., 2019	250	0.82	0.1860	201	0.82	0.1830	0.00	[-0.03; 0.04]	4.1%
Vos et al., 2019	278	0.55	0.0289	104	0.50	0.0150	0.05	[0.04; 0.05]	5.5%
Gupta et al., 2018	124	0.72	0.0330	44	0.68	0.0400	0.04	[0.03; 0.05]	5.3%
Kirichenko et al., 2018	25	0.65	0.1220	11	0.65	0.0630	-0.00	[-0.06; 0.06]	2.6%
Vohra et al., 2017	45	0.62	0.0800	30	0.53	0.0500	0.09	[0.06; 0.12]	4.4%
Fourie et al., 2015	66	0.63	0.0200	78	0.64	0.0100	-0.01	[-0.02; -0.00]	5.5%
Gleason et al., 2015	230	0.49	0.0214	51	0.47	0.0175	0.02	[0.01; 0.02]	5.5%
Zhu et al., 2014	50	0.58	0.1210	50	0.57	0.0946	0.01	[-0.03; 0.05]	3.5%
Suparna et al., 2013	26	0.63	0.2300	16	0.59	0.1700	0.04	[-0.08; 0.16]	1.0%
Brand et al., 2012	12	0.74	0.1000	13	0.76	0.1400	-0.02	[-0.11; 0.07]	1.4%
Cristofaro et al., 2011	222	1.00	0.2300	64	0.82	0.1300	0.18	[0.14; 0.22]	3.5%
Manfredi et al., 2011	16	1.30	0.6477	11	0.91	0.4983	0.40	[-0.04; 0.83]	0.1%
Calza et al., 2009	45	1.32	0.6971	21	0.89	0.5110	0.43	[0.13; 0.73]	0.2%
Seminari et al., 2002	28	0.67	0.1200	15	0.45	0.1200	0.22	[0.14; 0.30]	2.0%
**Random effects model**	**2346**			**1077**			**0.03**	**[0.02; 0.05]**	**71.1%**
Heterogeneity: $I^2 = 97.3\%$, $\tau^2 = 0.0008$, $p < 0.0001$									
**subgroup = Female**									
Chikwati et al., 2024	240	0.56	0.0200	61	0.58	0.0325	-0.02	[-0.03; -0.01]	5.4%
Parinello et al., 2012	374	0.73	0.1154	227	0.71	0.1114	0.02	[-0.00; 0.04]	5.0%
**Random effects model**	**614**			**288**			**-0.00**	**[-0.04; 0.03]**	**10.5%**
Heterogeneity: $I^2 = 92.2\%$, $\tau^2 = 0.0006$, $p = 0.0003$									
**subgroup = Male**									
Goulenok et al., 2015	30	0.76	0.0780	29	0.73	0.0830	0.03	[-0.01; 0.07]	3.6%
Pirs et al., 2014	60	0.71	0.1540	26	0.64	0.1100	0.07	[0.01; 0.13]	2.7%
Desvarieux et al., 2013	50	0.76	0.0900	50	0.73	0.0780	0.03	[-0.00; 0.06]	4.1%
van Vonderen et al., 2009	55	0.70	0.0140	22	0.66	0.0230	0.04	[0.03; 0.05]	5.4%
**Random effects model**	**195**			**127**			**0.04**	**[0.03; 0.05]**	**15.9%**
Heterogeneity: $I^2 = 0\%$, $\tau^2 = 0$, $p = 0.6463$									
**Random effects model**	**3205**			**1542**			**0.03**	**[0.02; 0.04]**	**100.0%**

Heterogeneity: $I^2 = 96.9\%$, $\tau^2 = 0.0008$, $p < 0.0001$
Test for overall effect: $z = 4.67$ ($p < 0.0001$)
Test for subgroup differences: $\chi^2_3 = 4.87$, df = 3 ($p = 0.1817$)

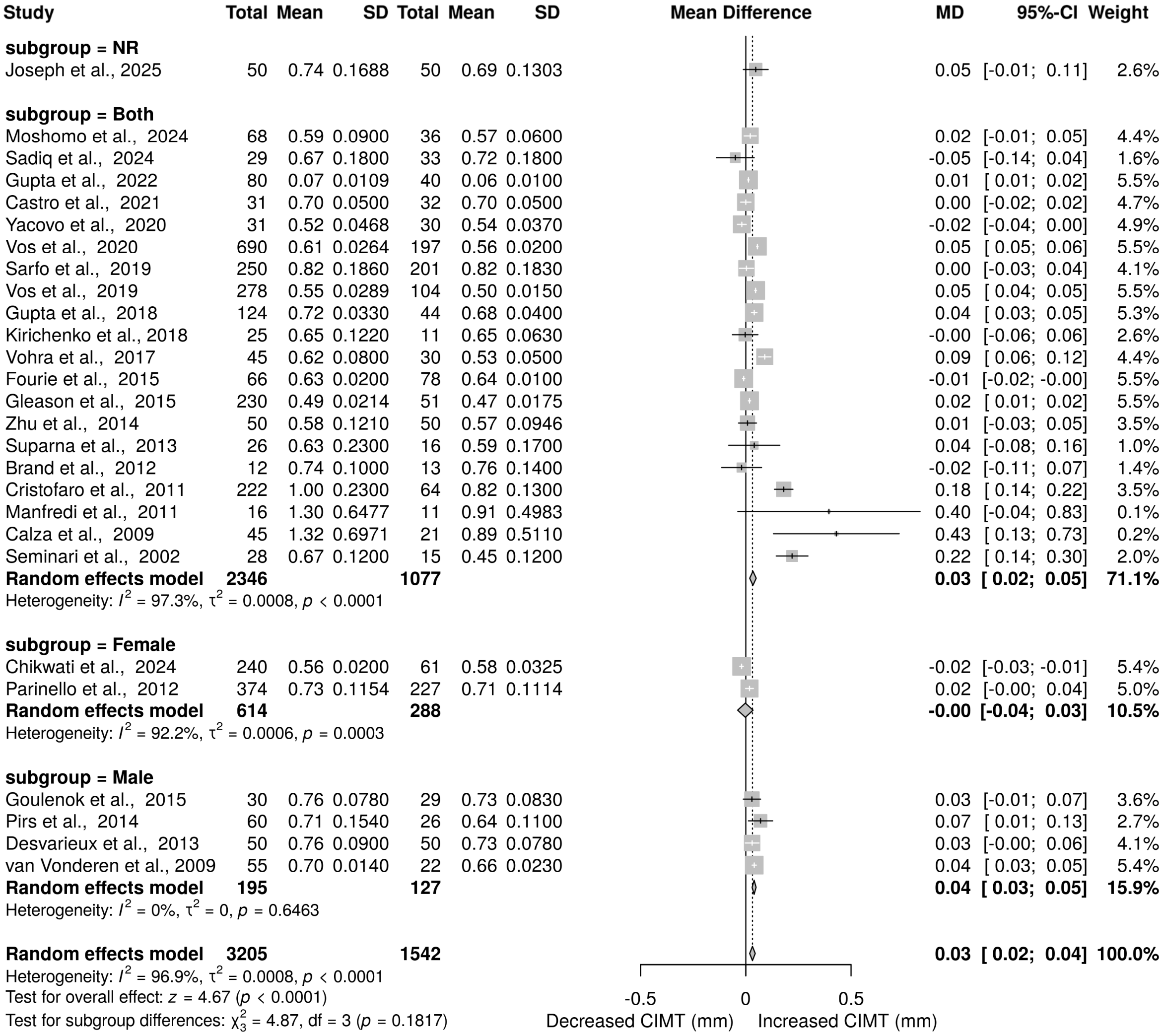

C

Study	PLHIV on ART Total	PLHIV on ART Mean	PLHIV on ART SD	ART naive PLHIV Total	ART naive PLHIV Mean	ART naive PLHIV SD	MD	95%-CI	Weight
**subgroup = Cross-sectional**									
Joseph et al., 2025	50	0.74	0.1688	50	0.69	0.1303	0.05	[-0.01; 0.11]	2.6%
Moshomo et al., 2024	68	0.59	0.0900	36	0.57	0.0600	0.02	[-0.01; 0.05]	4.4%
Sadiq et al., 2024	29	0.67	0.1800	33	0.72	0.1800	-0.05	[-0.14; 0.04]	1.6%
Chikwati et al., 2024	240	0.56	0.0200	61	0.58	0.0325	-0.02	[-0.03; -0.01]	5.4%
Gupta et al., 2022	80	0.07	0.0109	40	0.06	0.0100	0.01	[0.01; 0.02]	5.5%
Castro et al., 2021	31	0.70	0.0500	32	0.70	0.0500	0.00	[-0.02; 0.02]	4.7%
Vos et al., 2020	690	0.61	0.0264	197	0.56	0.0200	0.05	[0.05; 0.06]	5.5%
Sarfo et al., 2019	250	0.82	0.1860	201	0.82	0.1830	0.00	[-0.03; 0.04]	4.1%
Vos et al., 2019	278	0.55	0.0289	104	0.50	0.0150	0.05	[0.04; 0.05]	5.5%
Gupta et al., 2018	124	0.72	0.0330	44	0.68	0.0400	0.04	[0.03; 0.05]	5.3%
Kirichenko et al., 2018	25	0.65	0.1220	11	0.65	0.0630	-0.00	[-0.06; 0.06]	2.6%
Vohra et al., 2017	45	0.62	0.0800	30	0.53	0.0500	0.09	[0.06; 0.12]	4.4%
Goulenok et al., 2015	30	0.76	0.0780	29	0.73	0.0830	0.03	[-0.01; 0.07]	3.6%
Gleason et al., 2015	230	0.49	0.0214	51	0.47	0.0175	0.02	[0.01; 0.02]	5.5%
Pirs et al., 2014	60	0.71	0.1540	26	0.64	0.1100	0.07	[0.01; 0.13]	2.7%
Zhu et al., 2014	50	0.58	0.1210	50	0.57	0.0946	0.01	[-0.03; 0.05]	3.5%
Suparna et al., 2013	26	0.63	0.2300	16	0.59	0.1700	0.04	[-0.08; 0.16]	1.0%
Desvarieux et al., 2013	50	0.76	0.0900	50	0.73	0.0780	0.03	[-0.00; 0.06]	4.1%
Brand et al., 2012	12	0.74	0.1000	13	0.76	0.1400	-0.02	[-0.11; 0.07]	1.4%
Cristofaro et al., 2011	222	1.00	0.2300	64	0.82	0.1300	0.18	[0.14; 0.22]	3.5%
Manfredi et al., 2011	16	1.30	0.6477	11	0.91	0.4983	0.40	[-0.04; 0.83]	0.1%
Calza et al., 2009	45	1.32	0.6971	21	0.89	0.5110	0.43	[0.13; 0.73]	0.2%
Seminari et al., 2002	28	0.67	0.1200	15	0.45	0.1200	0.22	[0.14; 0.30]	2.0%
**Random effects model**	**2679**			**1185**			**0.04**	**[0.02; 0.05]**	**79.2%**
Heterogeneity: $I^2 = 96.3\%$, $\tau^2 = 0.0007$, $p < 0.0001$									
**subgroup = Cohort**									
Yacovo et al., 2020	31	0.52	0.0468	30	0.54	0.0370	-0.02	[-0.04; 0.00]	4.9%
Fourie et al., 2015	66	0.63	0.0200	78	0.64	0.0100	-0.01	[-0.02; -0.00]	5.5%
Parinello et al., 2012	374	0.73	0.1154	227	0.71	0.1114	0.02	[-0.00; 0.04]	5.0%
**Random effects model**	**471**			**335**			**-0.00**	**[-0.02; 0.01]**	**15.4%**
Heterogeneity: $I^2 = 77.1\%$, $\tau^2 = 0.0002$, $p = 0.0126$									
**subgroup = Case-control**									
van Vonderen et al., 2009	55	0.70	0.0140	22	0.66	0.0230	0.04	[0.03; 0.05]	5.4%
**Random effects model**	**3205**			**1542**			**0.03**	**[0.02; 0.04]**	**100.0%**

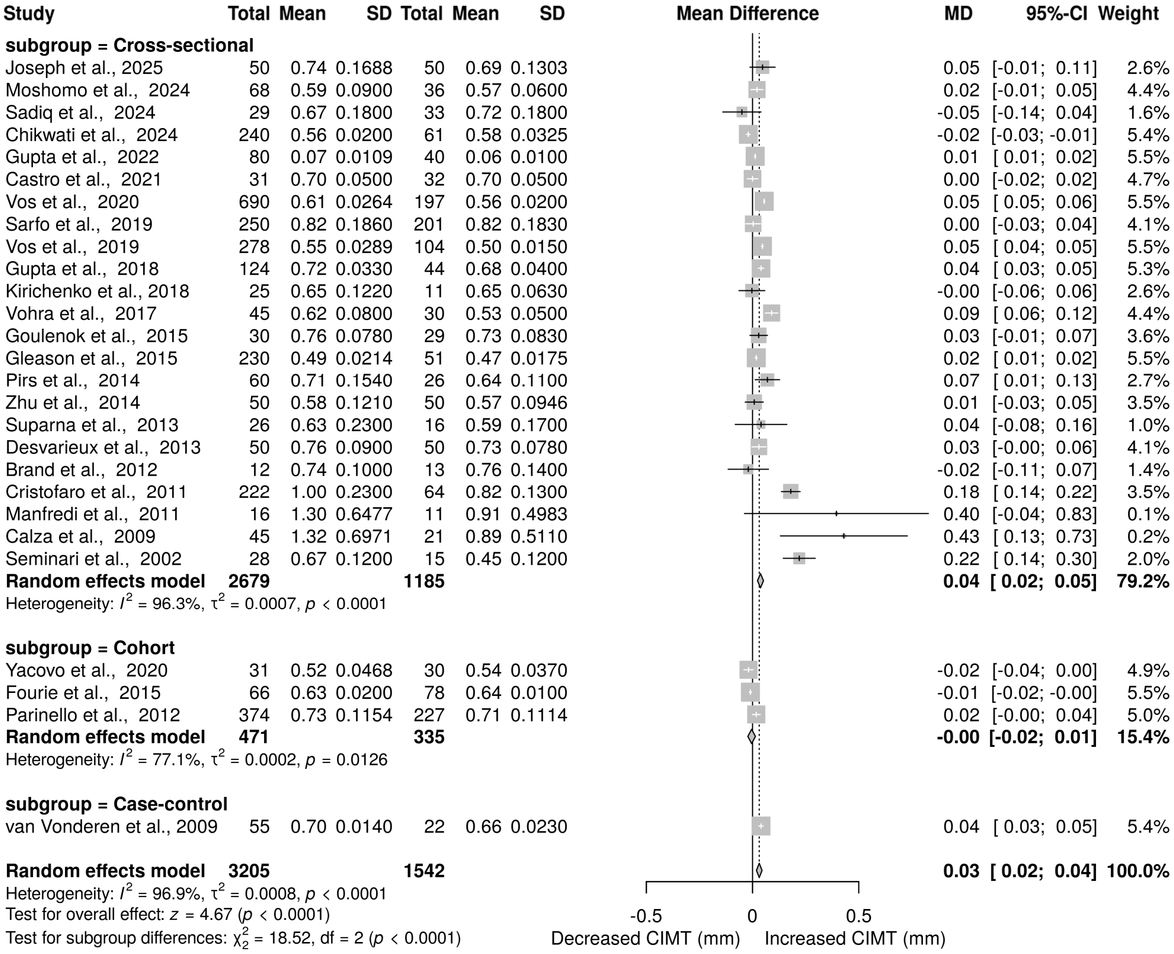

Heterogeneity: $I^2 = 96.9\%$, $\tau^2 = 0.0008$, $p < 0.0001$
Test for overall effect: $z = 4.67$ ($p < 0.0001$)
Test for subgroup differences: $\chi^2_2 = 18.52$, df = 2 ($p < 0.0001$)

D

Study	PLHIV on ART Total	PLHIV on ART Mean	PLHIV on ART SD	ART naive PLHIV Total	ART naive PLHIV Mean	ART naive PLHIV SD	MD	95%-CI	Weight
**subgroup = Equal to 100**									
Joseph et al., 2025	50	0.74	0.1688	50	0.69	0.1303	0.05	[-0.01; 0.11]	2.6%
Zhu et al., 2014	50	0.58	0.1210	50	0.57	0.0946	0.01	[-0.03; 0.05]	3.5%
Desvarieux et al., 2013	50	0.76	0.0900	50	0.73	0.0780	0.03	[-0.00; 0.06]	4.1%
**Random effects model**	**150**			**150**			**0.03**	**[0.00; 0.05]**	**10.3%**
Heterogeneity: $I^2 = 0\%$, $\tau^2 = 0$, $p = 0.5407$									
**subgroup = Above 100**									
Moshomo et al., 2024	68	0.59	0.0900	36	0.57	0.0600	0.02	[-0.01; 0.05]	4.4%
Chikwati et al., 2024	240	0.56	0.0200	61	0.58	0.0325	-0.02	[-0.03; -0.01]	5.4%
Gupta et al., 2022	80	0.07	0.0109	40	0.06	0.0100	0.01	[0.01; 0.02]	5.5%
Vos et al., 2020	690	0.61	0.0264	197	0.56	0.0200	0.05	[0.05; 0.06]	5.5%
Sarfo et al., 2019	250	0.82	0.1860	201	0.82	0.1830	0.00	[-0.03; 0.04]	4.1%
Vos et al., 2019	278	0.55	0.0289	104	0.50	0.0150	0.05	[0.04; 0.05]	5.5%
Gupta et al., 2018	124	0.72	0.0330	44	0.68	0.0400	0.04	[0.03; 0.05]	5.3%
Fourie et al., 2015	66	0.63	0.0200	78	0.64	0.0100	-0.01	[-0.02; -0.00]	5.5%
Gleason et al., 2015	230	0.49	0.0214	51	0.47	0.0175	0.02	[0.01; 0.02]	5.5%
Parinello et al., 2012	374	0.73	0.1154	227	0.71	0.1114	0.02	[-0.00; 0.04]	5.0%
Cristofaro et al., 2011	222	1.00	0.2300	64	0.82	0.1300	0.18	[0.14; 0.22]	3.5%
**Random effects model**	**2622**			**1103**			**0.03**	**[0.01; 0.05]**	**55.3%**
Heterogeneity: $I^2 = 98.7\%$, $\tau^2 = 0.0008$, $p < 0.0001$									
**subgroup = Below 100**									
Sadiq et al., 2024	29	0.67	0.1800	33	0.72	0.1800	-0.05	[-0.14; 0.04]	1.6%
Castro et al., 2021	31	0.70	0.0500	32	0.70	0.0500	0.00	[-0.02; 0.02]	4.7%
Yacovo et al., 2020	31	0.52	0.0468	30	0.54	0.0370	-0.02	[-0.04; 0.00]	4.9%
Kirichenko et al., 2018	25	0.65	0.1220	11	0.65	0.0630	-0.00	[-0.06; 0.06]	2.6%
Vohra et al., 2017	45	0.62	0.0800	30	0.53	0.0500	0.09	[0.06; 0.12]	4.4%
Goulenok et al., 2015	30	0.76	0.0780	29	0.73	0.0830	0.03	[-0.01; 0.07]	3.6%
Pirs et al., 2014	60	0.71	0.1540	26	0.64	0.1100	0.07	[0.01; 0.13]	2.7%
Suparna et al., 2013	26	0.63	0.2300	16	0.59	0.1700	0.04	[-0.08; 0.16]	1.0%
Brand et al., 2012	12	0.74	0.1000	13	0.76	0.1400	-0.02	[-0.11; 0.07]	1.4%
Manfredi et al., 2011	16	1.30	0.6477	11	0.91	0.4983	0.40	[-0.04; 0.83]	0.1%
Calza et al., 2009	45	1.32	0.6971	21	0.89	0.5110	0.43	[0.13; 0.73]	0.2%
van Vonderen et al., 2009	55	0.70	0.0140	22	0.66	0.0230	0.04	[0.03; 0.05]	5.4%
Seminari et al., 2002	28	0.67	0.1200	15	0.45	0.1200	0.22	[0.14; 0.30]	2.0%
**Random effects model**	**433**			**289**			**0.04**	**[0.01; 0.07]**	**34.4%**
Heterogeneity: $I^2 = 86.2\%$, $\tau^2 = 0.0019$, $p < 0.0001$									
**Random effects model**	**3205**			**1542**			**0.03**	**[0.02; 0.04]**	**100.0%**

Heterogeneity: $I^2 = 96.9\%$, $\tau^2 = 0.0008$, $p < 0.0001$
Test for overall effect: $z = 4.67$ ($p < 0.0001$)
Test for subgroup differences: $\chi^2_2 = 0.66$, df = 2 ($p = 0.7205$)

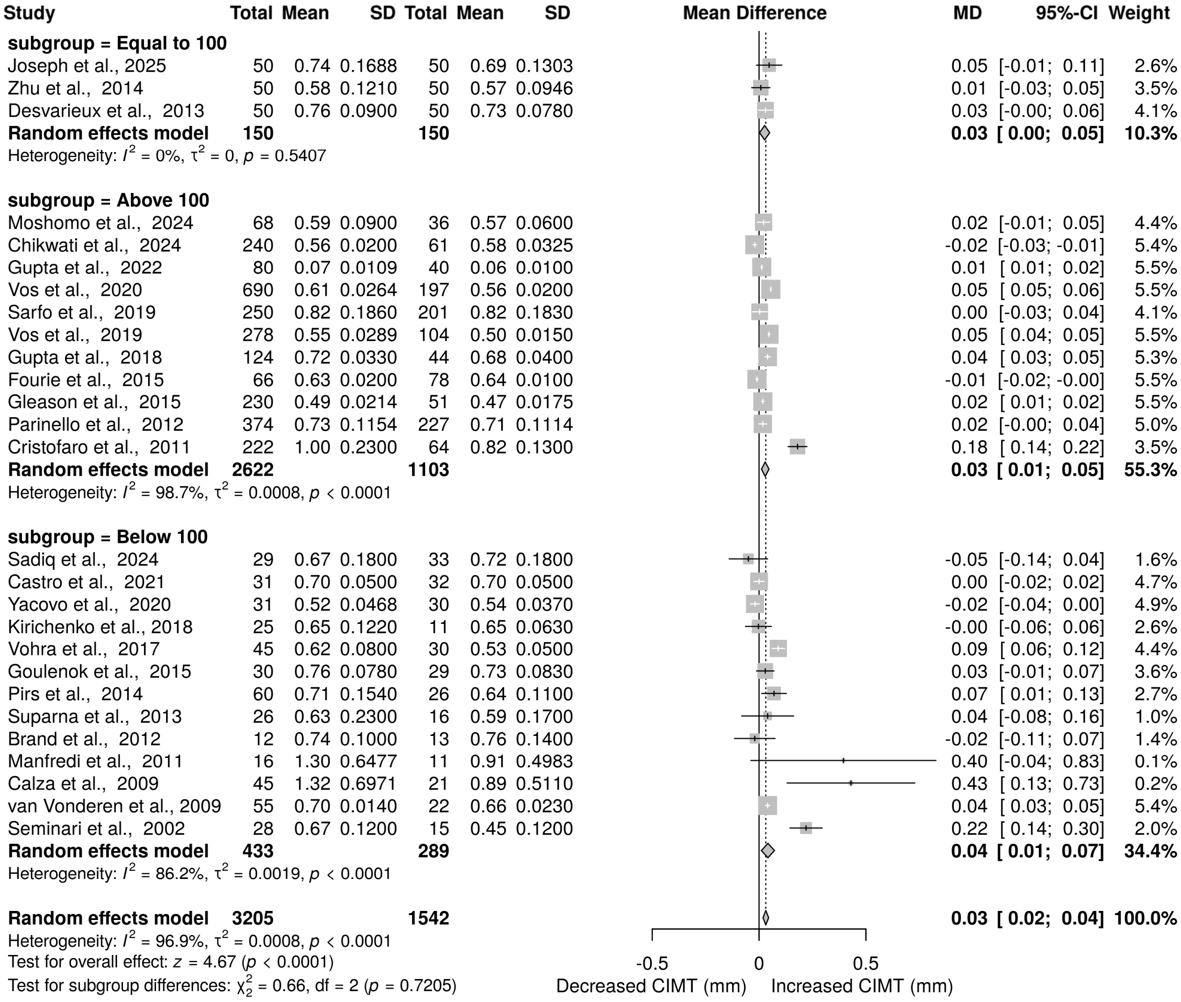

E

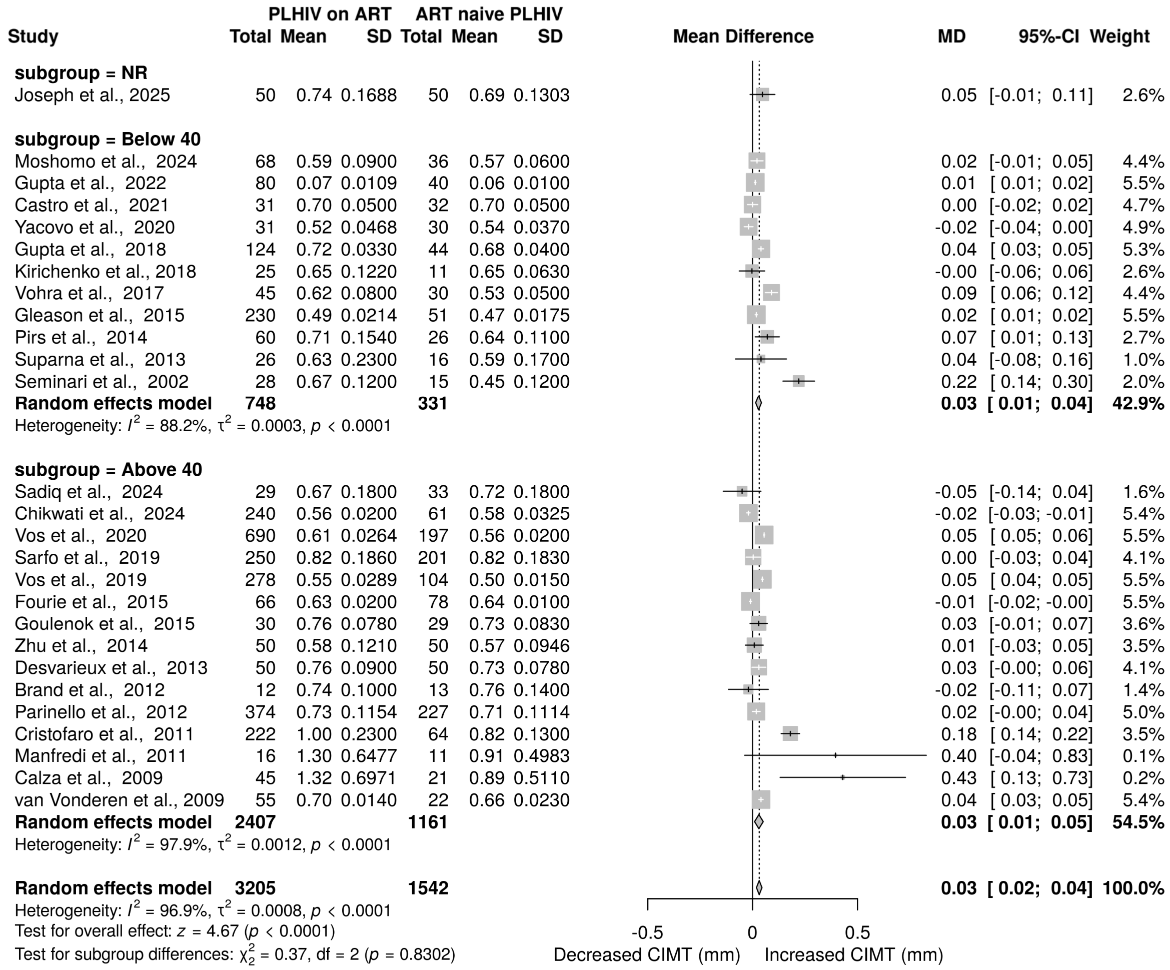
PLHIV on ART ART naive PLHIV
Study Total Mean SD Total Mean SD Mean Difference MD 95%-CI Weight
subgroup = NR
Joseph et al., 2025 50 0.74 0.1688 50 0.69 0.1303 0.05 [-0.01; 0.11] 2.6%
subgroup = Below 40
Moshomo et al., 2024 68 0.59 0.0900 36 0.57 0.0600 0.02 [-0.01; 0.05] 4.4%
Gupta et al., 2022 80 0.07 0.0109 40 0.06 0.0100 0.01 [0.01; 0.02] 5.5%
Castro et al., 2021 31 0.70 0.0500 32 0.70 0.0500 0.00 [-0.02; 0.02] 4.7%
Yacovo et al., 2020 31 0.52 0.0468 30 0.54 0.0370 -0.02 [-0.04; 0.00] 4.9%
Gupta et al., 2018 124 0.72 0.0330 44 0.68 0.0400 0.04 [0.03; 0.05] 5.3%
Kirichenko et al., 2018 25 0.65 0.1220 11 0.65 0.0630 -0.00 [-0.06; 0.06] 2.6%
Vohra et al., 2017 45 0.62 0.0800 30 0.53 0.0500 0.09 [0.06; 0.12] 4.4%
Gleason et al., 2015 230 0.49 0.0214 51 0.47 0.0175 0.02 [0.01; 0.02] 5.5%
Pirs et al., 2014 60 0.71 0.1540 26 0.64 0.1100 0.07 [0.01; 0.13] 2.7%
Suparna et al., 2013 26 0.63 0.2300 16 0.59 0.1700 0.04 [-0.08; 0.16] 1.0%
Seminari et al., 2002 28 0.67 0.1200 15 0.45 0.1200 0.22 [0.14; 0.30] 2.0%
Random effects model 748 331 0.03 [0.01; 0.04] 42.9%
Heterogeneity: I^2 = 88.2%, τ^2 = 0.0003, p < 0.0001
subgroup = Above 40
Sadiq et al., 2024 29 0.67 0.1800 33 0.72 0.1800 -0.05 [-0.14; 0.04] 1.6%
Chikwati et al., 2024 240 0.56 0.0200 61 0.58 0.0325 -0.02 [-0.03; -0.01] 5.4%
Vos et al., 2020 690 0.61 0.0264 197 0.56 0.0200 0.05 [0.05; 0.06] 5.5%
Sarfo et al., 2019 250 0.82 0.1860 201 0.82 0.1830 0.00 [-0.03; 0.04] 4.1%
Vos et al., 2019 278 0.55 0.0289 104 0.50 0.0150 0.05 [0.04; 0.05] 5.5%
Fourie et al., 2015 66 0.63 0.0200 78 0.64 0.0100 -0.01 [-0.02; -0.00] 5.5%
Goulenok et al., 2015 30 0.76 0.0780 29 0.73 0.0830 0.03 [-0.01; 0.07] 3.6%
Zhu et al., 2014 50 0.58 0.1210 50 0.57 0.0946 0.01 [-0.03; 0.05] 3.5%
Desvarieux et al., 2013 50 0.76 0.0900 50 0.73 0.0780 0.03 [-0.00; 0.06] 4.1%
Brand et al., 2012 12 0.74 0.1000 13 0.76 0.1400 -0.02 [-0.11; 0.07] 1.4%
Parinello et al., 2012 374 0.73 0.1154 227 0.71 0.1114 0.02 [-0.00; 0.04] 5.0%
Cristofaro et al., 2011 222 1.00 0.2300 64 0.82 0.1300 0.18 [0.14; 0.22] 3.5%
Manfredi et al., 2011 16 1.30 0.6477 11 0.91 0.4983 0.40 [-0.04; 0.83] 0.1%
Calza et al., 2009 45 1.32 0.6971 21 0.89 0.5110 0.43 [0.13; 0.73] 0.2%
van Vonderen et al., 2009 55 0.70 0.0140 22 0.66 0.0230 0.04 [0.03; 0.05] 5.4%
Random effects model 2407 1161 0.03 [0.01; 0.05] 54.5%
Heterogeneity: I^2 = 97.9%, τ^2 = 0.0012, p < 0.0001
Random effects model 3205 1542 0.03 [0.02; 0.04] 100.0%
Heterogeneity: I^2 = 96.9%, τ^2 = 0.0008, p < 0.0001
Test for overall effect: z = 4.67 (p < 0.0001)
Test for subgroup differences: χ^2_2 = 0.37, df = 2 (p = 0.8302)
-0.5 0 0.5
Decreased CIMT (mm) Increased CIMT (mm)

F

Study	PLHIV on ART Total	PLHIV on ART Mean	PLHIV on ART SD	ART naive PLHIV Total	ART naive PLHIV Mean	ART naive PLHIV SD	MD	95%-CI	Weight
**subgroup = Low**									
Joseph et al., 2025	50	0.74	0.1688	50	0.69	0.1303	0.05	[-0.01; 0.11]	2.6%
Moshomo et al., 2024	68	0.59	0.0900	36	0.57	0.0600	0.02	[-0.01; 0.05]	4.4%
Sadiq et al., 2024	29	0.67	0.1800	33	0.72	0.1800	-0.05	[-0.14; 0.04]	1.6%
Chikwati et al., 2024	240	0.56	0.0200	61	0.58	0.0325	-0.02	[-0.03; -0.01]	5.4%
Yacovo et al., 2020	31	0.52	0.0468	30	0.54	0.0370	-0.02	[-0.04; 0.00]	4.9%
Vos et al., 2020	690	0.61	0.0264	197	0.56	0.0200	0.05	[0.05; 0.06]	5.5%
Sarfo et al., 2019	250	0.82	0.1860	201	0.82	0.1830	0.00	[-0.03; 0.04]	4.1%
Vos et al., 2019	278	0.55	0.0289	104	0.50	0.0150	0.05	[0.04; 0.05]	5.5%
Gupta et al., 2018	124	0.72	0.0330	44	0.68	0.0400	0.04	[0.03; 0.05]	5.3%
Kirichenko et al., 2018	25	0.65	0.1220	11	0.65	0.0630	-0.00	[-0.06; 0.06]	2.6%
Vohra et al., 2017	45	0.62	0.0800	30	0.53	0.0500	0.09	[0.06; 0.12]	4.4%
Fourie et al., 2015	66	0.63	0.0200	78	0.64	0.0100	-0.01	[-0.02; -0.00]	5.5%
Goulenok et al., 2015	30	0.76	0.0780	29	0.73	0.0830	0.03	[-0.01; 0.07]	3.6%
Gleason et al., 2015	230	0.49	0.0214	51	0.47	0.0175	0.02	[0.01; 0.02]	5.5%
Pirs et al., 2014	60	0.71	0.1540	26	0.64	0.1100	0.07	[0.01; 0.13]	2.7%
Zhu et al., 2014	50	0.58	0.1210	50	0.57	0.0946	0.01	[-0.03; 0.05]	3.5%
Desvarieux et al., 2013	50	0.76	0.0900	50	0.73	0.0780	0.03	[-0.00; 0.06]	4.1%
Brand et al., 2012	12	0.74	0.1000	13	0.76	0.1400	-0.02	[-0.11; 0.07]	1.4%
Parinello et al., 2012	374	0.73	0.1154	227	0.71	0.1114	0.02	[-0.00; 0.04]	5.0%
Cristofaro et al., 2011	222	1.00	0.2300	64	0.82	0.1300	0.18	[0.14; 0.22]	3.5%
Manfredi et al., 2011	16	1.30	0.6477	11	0.91	0.4983	0.40	[-0.04; 0.83]	0.1%
Calza et al., 2009	45	1.32	0.6971	21	0.89	0.5110	0.43	[0.13; 0.73]	0.2%
van Vonderen et al., 2009	55	0.70	0.0140	22	0.66	0.0230	0.04	[0.03; 0.05]	5.4%
Seminari et al., 2002	28	0.67	0.1200	15	0.45	0.1200	0.22	[0.14; 0.30]	2.0%
**Random effects model**	**3068**			**1454**			**0.03**	**[0.02; 0.05]**	**88.8%**
Heterogeneity: $I^2 = 97\%$, $\tau^2 = 0.0010$, $p < 0.0001$									
**subgroup = Moderate**									
Gupta et al., 2022	80	0.07	0.0109	40	0.06	0.0100	0.01	[0.01; 0.02]	5.5%
Castro et al., 2021	31	0.70	0.0500	32	0.70	0.0500	0.00	[-0.02; 0.02]	4.7%
Suparna et al., 2013	26	0.63	0.2300	16	0.59	0.1700	0.04	[-0.08; 0.16]	1.0%
**Random effects model**	**137**			**88**			**0.01**	**[0.01; 0.02]**	**11.2%**
Heterogeneity: $I^2 = 0\%$, $\tau^2 = 0$, $p = 0.5596$									
**Random effects model**	**3205**			**1542**			**0.03**	**[0.02; 0.04]**	**100.0%**

Heterogeneity: $I^2 = 96.9\%$, $\tau^2 = 0.0008$, $p < 0.0001$
Test for overall effect: $z = 4.67$ ($p < 0.0001$)
Test for subgroup differences: $\chi^2_1 = 8.14$, df = 1 ($p = 0.0043$)

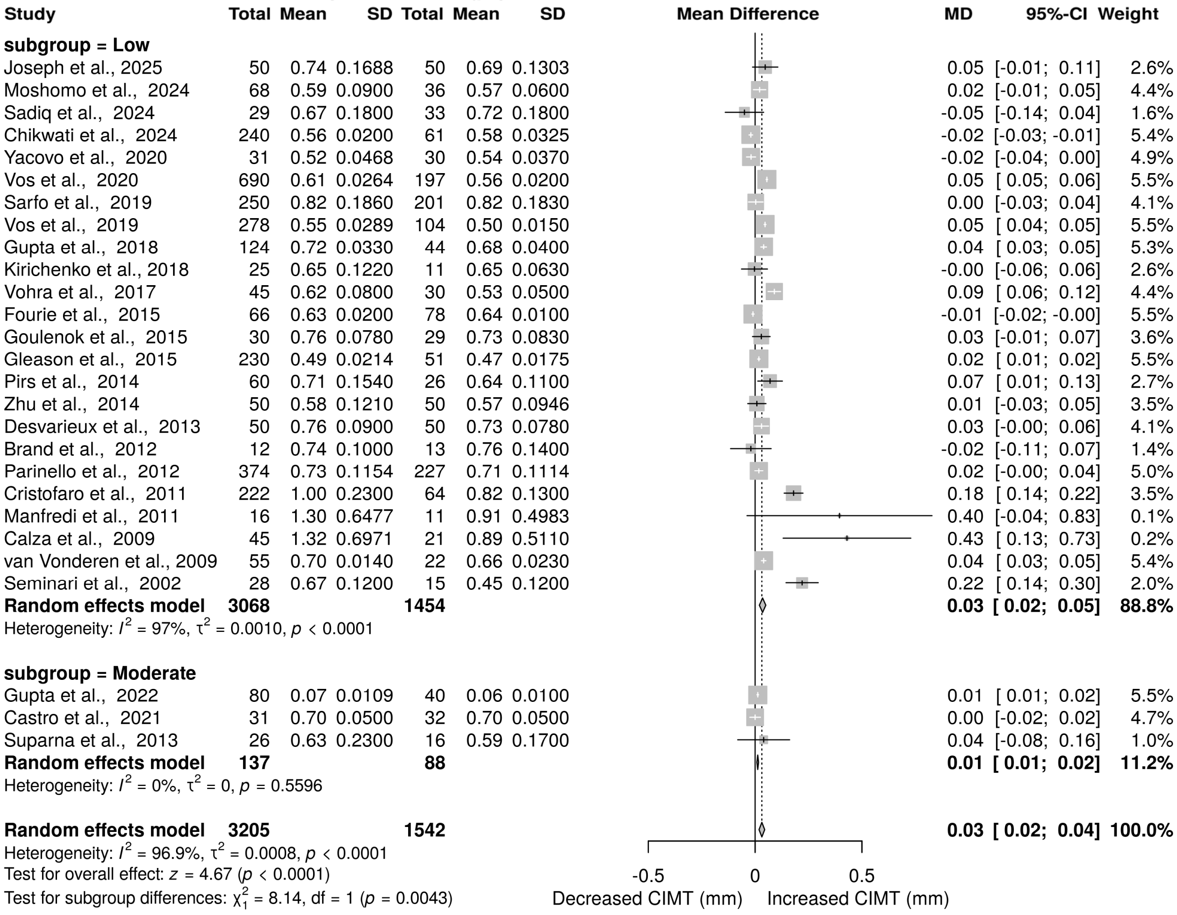

G

Study	PLHIV on ART Total	PLHIV on ART Mean	PLHIV on ART SD	ART naive PLHIV Total	ART naive PLHIV Mean	ART naive PLHIV SD	MD	95%-CI	Weight
**subgroup = Not Reported**									
Joseph et al., 2025	50	0.74	0.1688	50	0.69	0.1303	0.05	[-0.01; 0.11]	2.6%
Sarfo et al., 2019	250	0.82	0.1860	201	0.82	0.1830	0.00	[-0.03; 0.04]	4.1%
Kirichenko et al., 2018	25	0.65	0.1220	11	0.65	0.0630	-0.00	[-0.06; 0.06]	2.6%
Gleason et al., 2015	230	0.49	0.0214	51	0.47	0.0175	0.02	[0.01; 0.02]	5.5%
Pirs et al., 2014	60	0.71	0.1540	26	0.64	0.1100	0.07	[0.01; 0.13]	2.7%
Suparna et al., 2013	26	0.63	0.2300	16	0.59	0.1700	0.04	[-0.08; 0.16]	1.0%
Brand et al., 2012	12	0.74	0.1000	13	0.76	0.1400	-0.02	[-0.11; 0.07]	1.4%
Seminari et al., 2002	28	0.67	0.1200	15	0.45	0.1200	0.22	[0.14; 0.30]	2.0%
**Random effects model**	**681**			**383**			**0.04**	**[0.01; 0.08]**	**21.9%**
Heterogeneity: $I^2 = 79.2\%$, $\tau^2 = 0.0017$, $p < 0.0001$									
**subgroup = Pre-Rapid ART Initiation**									
Moshomo et al., 2024	68	0.59	0.0900	36	0.57	0.0600	0.02	[-0.01; 0.05]	4.4%
Yacovo et al., 2020	31	0.52	0.0468	30	0.54	0.0370	-0.02	[-0.04; 0.00]	4.9%
Gupta et al., 2018	124	0.72	0.0330	44	0.68	0.0400	0.04	[0.03; 0.05]	5.3%
Fourie et al., 2015	66	0.63	0.0200	78	0.64	0.0100	-0.01	[-0.02; -0.00]	5.5%
Goulenok et al., 2015	30	0.76	0.0780	29	0.73	0.0830	0.03	[-0.01; 0.07]	3.6%
Zhu et al., 2014	50	0.58	0.1210	50	0.57	0.0946	0.01	[-0.03; 0.05]	3.5%
Desvarieux et al., 2013	50	0.76	0.0900	50	0.73	0.0780	0.03	[-0.00; 0.06]	4.1%
Parinello et al., 2012	374	0.73	0.1154	227	0.71	0.1114	0.02	[-0.00; 0.04]	5.0%
Cristofaro et al., 2011	222	1.00	0.2300	64	0.82	0.1300	0.18	[0.14; 0.22]	3.5%
Manfredi et al., 2011	16	1.30	0.6477	11	0.91	0.4983	0.40	[-0.04; 0.83]	0.1%
Calza et al., 2009	45	1.32	0.6971	21	0.89	0.5110	0.43	[0.13; 0.73]	0.2%
van Vonderen et al., 2009	55	0.70	0.0140	22	0.66	0.0230	0.04	[0.03; 0.05]	5.4%
**Random effects model**	**1131**			**662**			**0.03**	**[0.01; 0.06]**	**45.5%**
Heterogeneity: $I^2 = 94\%$, $\tau^2 = 0.0012$, $p < 0.0001$									
**subgroup = Rapid ART Initiation**									
Sadiq et al., 2024	29	0.67	0.1800	33	0.72	0.1800	-0.05	[-0.14; 0.04]	1.6%
Chikwati et al., 2024	240	0.56	0.0200	61	0.58	0.0325	-0.02	[-0.03; -0.01]	5.4%
Gupta et al., 2022	80	0.07	0.0109	40	0.06	0.0100	0.01	[0.01; 0.02]	5.5%
Castro et al., 2021	31	0.70	0.0500	32	0.70	0.0500	0.00	[-0.02; 0.02]	4.7%
Vos et al., 2020	690	0.61	0.0264	197	0.56	0.0200	0.05	[0.05; 0.06]	5.5%
Vos et al., 2019	278	0.55	0.0289	104	0.50	0.0150	0.05	[0.04; 0.05]	5.5%
Vohra et al., 2017	45	0.62	0.0800	30	0.53	0.0500	0.09	[0.06; 0.12]	4.4%
**Random effects model**	**1393**			**497**			**0.03**	**[0.00; 0.05]**	**32.6%**
Heterogeneity: $I^2 = 98.7\%$, $\tau^2 = 0.0008$, $p < 0.0001$									
**Random effects model**	**3205**			**1542**			**0.03**	**[0.02; 0.04]**	**100.0%**
Heterogeneity: $I^2 = 96.9\%$, $\tau^2 = 0.0008$, $p < 0.0001$									
Test for overall effect: $z = 4.67$ ($p < 0.0001$)									
Test for subgroup differences: $\chi^2_2 = 0.68$, df = 2 ($p = 0.7134$)									

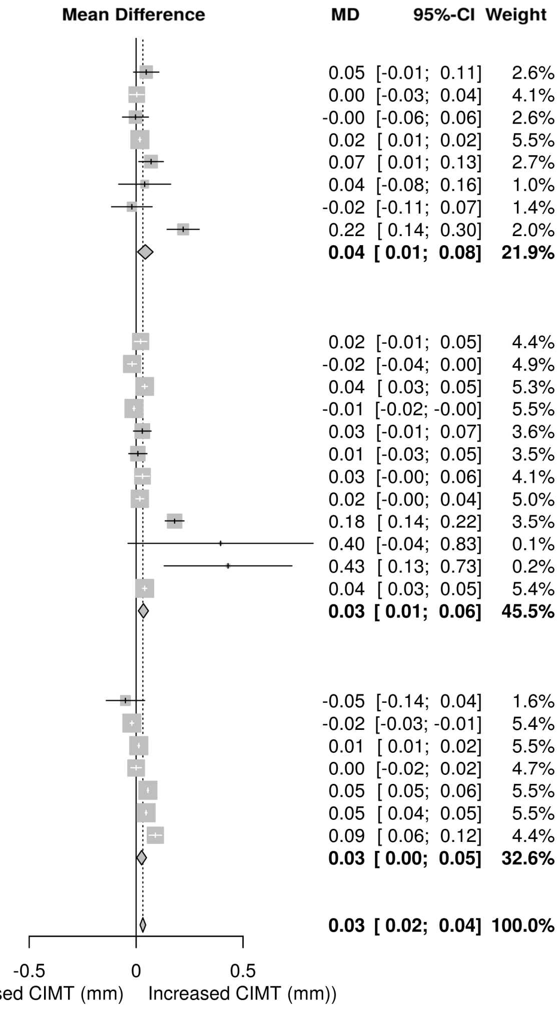

H

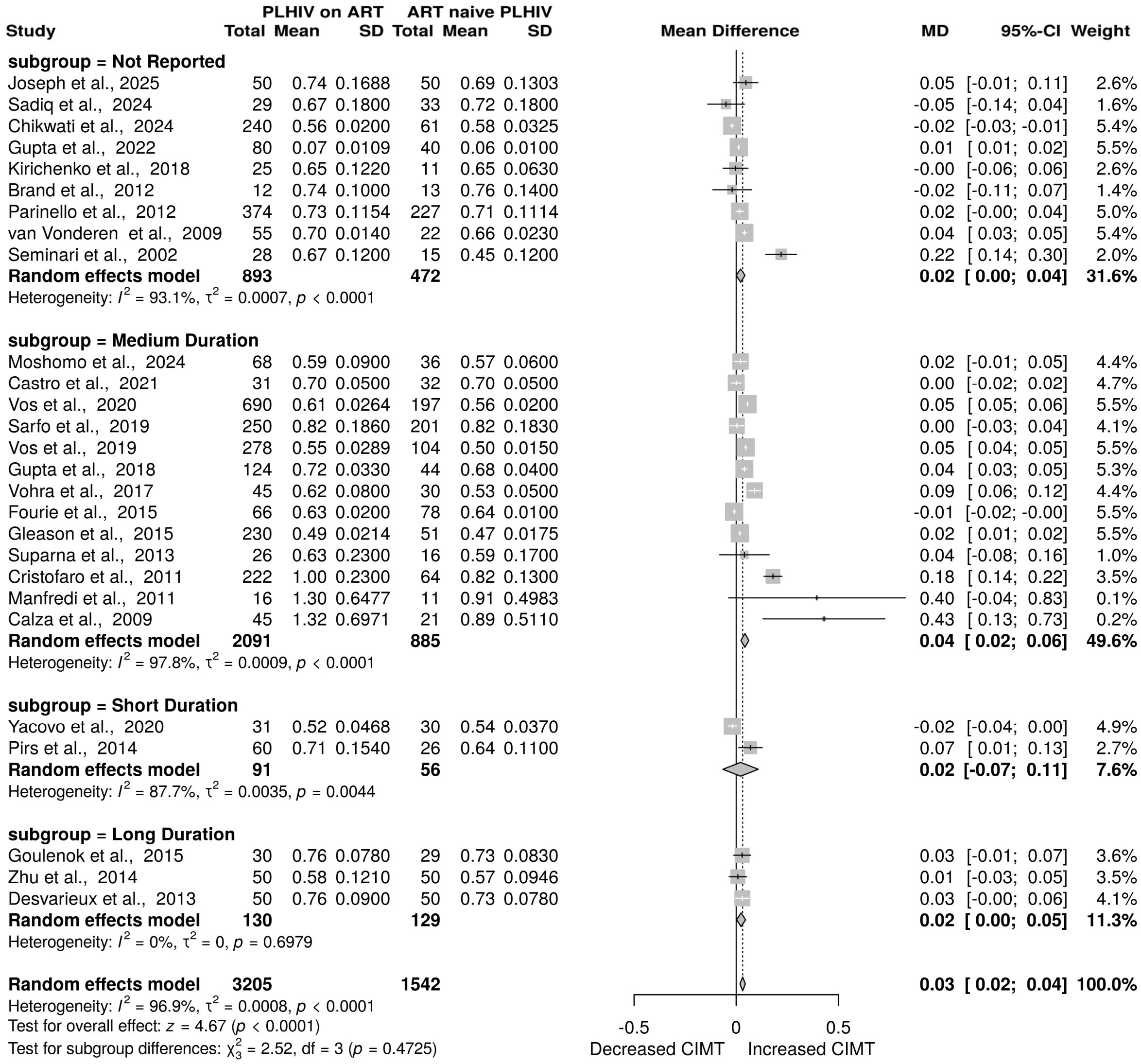
PLHIV on ART ART naive PLHIV
Study Total Mean SD Total Mean SD Mean Difference MD 95%-CI Weight
subgroup = Not Reported
Joseph et al., 2025 50 0.74 0.1688 50 0.69 0.1303 0.05 [-0.01; 0.11] 2.6%
Sadiq et al., 2024 29 0.67 0.1800 33 0.72 0.1800 -0.05 [-0.14; 0.04] 1.6%
Chikwati et al., 2024 240 0.56 0.0200 61 0.58 0.0325 -0.02 [-0.03; -0.01] 5.4%
Gupta et al., 2022 80 0.07 0.0109 40 0.06 0.0100 0.01 [0.01; 0.02] 5.5%
Kirichenko et al., 2018 25 0.65 0.1220 11 0.65 0.0630 -0.00 [-0.06; 0.06] 2.6%
Brand et al., 2012 12 0.74 0.1000 13 0.76 0.1400 -0.02 [-0.11; 0.07] 1.4%
Parinello et al., 2012 374 0.73 0.1154 227 0.71 0.1114 0.02 [-0.00; 0.04] 5.0%
van Vonderen et al., 2009 55 0.70 0.0140 22 0.66 0.0230 0.04 [0.03; 0.05] 5.4%
Seminari et al., 2002 28 0.67 0.1200 15 0.45 0.1200 0.22 [0.14; 0.30] 2.0%
Random effects model 893 472 0.02 [0.00; 0.04] 31.6%
Heterogeneity: I^2 = 93.1%, τ^2 = 0.0007, p < 0.0001
subgroup = Medium Duration
Moshomo et al., 2024 68 0.59 0.0900 36 0.57 0.0600 0.02 [-0.01; 0.05] 4.4%
Castro et al., 2021 31 0.70 0.0500 32 0.70 0.0500 0.00 [-0.02; 0.02] 4.7%
Vos et al., 2020 690 0.61 0.0264 197 0.56 0.0200 0.05 [0.05; 0.06] 5.5%
Sarfo et al., 2019 250 0.82 0.1860 201 0.82 0.1830 0.00 [-0.03; 0.04] 4.1%
Vos et al., 2019 278 0.55 0.0289 104 0.50 0.0150 0.05 [0.04; 0.05] 5.5%
Gupta et al., 2018 124 0.72 0.0330 44 0.68 0.0400 0.04 [0.03; 0.05] 5.3%
Vohra et al., 2017 45 0.62 0.0800 30 0.53 0.0500 0.09 [0.06; 0.12] 4.4%
Fourie et al., 2015 66 0.63 0.0200 78 0.64 0.0100 -0.01 [-0.02; -0.00] 5.5%
Gleason et al., 2015 230 0.49 0.0214 51 0.47 0.0175 0.02 [0.01; 0.02] 5.5%
Suparna et al., 2013 26 0.63 0.2300 16 0.59 0.1700 0.04 [-0.08; 0.16] 1.0%
Cristofaro et al., 2011 222 1.00 0.2300 64 0.82 0.1300 0.18 [0.14; 0.22] 3.5%
Manfredi et al., 2011 16 1.30 0.6477 11 0.91 0.4983 0.40 [-0.04; 0.83] 0.1%
Calza et al., 2009 45 1.32 0.6971 21 0.89 0.5110 0.43 [0.13; 0.73] 0.2%
Random effects model 2091 885 0.04 [0.02; 0.06] 49.6%
Heterogeneity: I^2 = 97.8%, τ^2 = 0.0009, p < 0.0001
subgroup = Short Duration
Yacovo et al., 2020 31 0.52 0.0468 30 0.54 0.0370 -0.02 [-0.04; 0.00] 4.9%
Pirs et al., 2014 60 0.71 0.1540 26 0.64 0.1100 0.07 [0.01; 0.13] 2.7%
Random effects model 91 56 0.02 [-0.07; 0.11] 7.6%
Heterogeneity: I^2 = 87.7%, τ^2 = 0.0035, p = 0.0044
subgroup = Long Duration
Goulenok et al., 2015 30 0.76 0.0780 29 0.73 0.0830 0.03 [-0.01; 0.07] 3.6%
Zhu et al., 2014 50 0.58 0.1210 50 0.57 0.0946 0.01 [-0.03; 0.05] 3.5%
Desvarieux et al., 2013 50 0.76 0.0900 50 0.73 0.0780 0.03 [-0.00; 0.06] 4.1%
Random effects model 130 129 0.02 [0.00; 0.05] 11.3%
Heterogeneity: I^2 = 0%, τ^2 = 0, p = 0.6979
Random effects model 3205 1542 0.03 [0.02; 0.04] 100.0%
Heterogeneity: I^2 = 96.9%, τ^2 = 0.0008, p < 0.0001
Test for overall effect: z = 4.67 (p < 0.0001)
Test for subgroup differences: χ^2_3 = 2.52, df = 3 (p = 0.4725)
-0.5 0 0.5
Decreased CIMT Increased CIMT

I

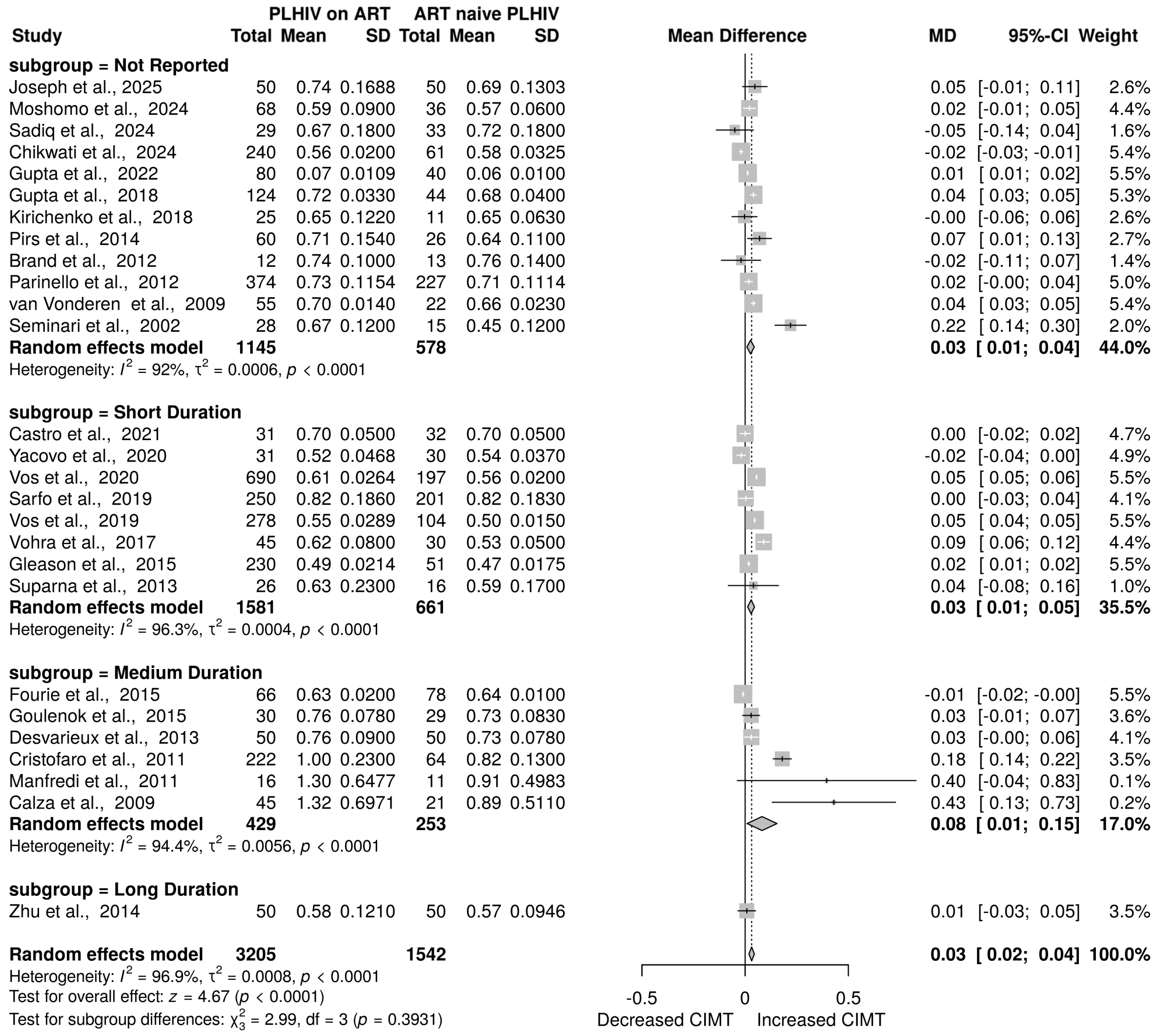

J

Figure S1: Subgroup analysis for CIMT in ART compared to ART-naïve. A. Subgroup analysis for CIMT in ART compared to ART-naïve based on continent of publication. B. Subgroup analysis for CIMT in ART compared to ART-naïve based on CD4 count for patients on ART. C. Subgroup analysis for CIMT in ART compared to ART naive patients based on gender. D. Subgroup analysis for CIMT in ART compared to ART naive patients based on study design. E. Subgroup analysis for CIMT in ART compared to ART naive patients based on sample size. F. Subgroup analysis for CIMT in ART compared to ART naive patients based on age. G. Subgroup analysis for CIMT in ART compared to ART naive patients based on the risk of bias. H. Subgroup analysis for CIMT in ART compared to ART naive patients based on WHO adoption of rapid ART initiation. I. Subgroup analysis based on the duration of HIV infection since diagnosis in PLHIV on ART. J. Subgroup analysis based on the duration of HIV infection since diagnosis in PLHIV on ART naive patients.

Table S2: Sensitivity analysis, showing the effect of the exclusion of one study on CIMT in PLHIV on ART compared to ART-naïve

		95% Confidence Intervals			
**Excluded study**	**Effect size**	**Lower**	**Upper**	**p**	**I^2**
Joseph et al.,	0.03	0.02	0.04	< 0.0001	97
Moshomo et al.,	0.03	0.02	0.05	< 0.0001	97
Sadiq et al.,	0.03	0.02	0.05	< 0.0001	97
Chikwati et al.,	0.03	0.02	0.05	<0.0001	96.5
Gupta et al.,	0.03	0.02	0.05	<0.0001	96.7
Castro et al.,	0.03	0.02	0.05	< 0.0001	97
Yacovo et al.,	0.03	0.02	0.05	< 0.0001	97
Vos et al.,	0.03	0.02	0.04	<0.0001	95.2
Sarfo et al.,	0.03	0.02	0.05	< 0.0001	97
Vos et al.,	0.03	0.02	0.05	<0.0001	96.7
Gupta et al.,	0.03	0.02	0.04	<0.0001	97
Kirichenko et al.,	0.03	0.02	0.05	< 0.0001	97
Vohra et al.,	0.03	0.02	0.04	<0.0001	97
Fourie et al.,	0.03	0.02	0.05	< 0.0001	96
Goulenok et al.,	0.03	0.02	0.05	<0.0001	97
Pirs et al.,	0.03	0.02	0.05	<0.0001	97
Zhu et al.,	0.03	0.02	0.04	< 0.0001	97
Gleason et al.,	0.03	0.02	0.05	< 0.0001	97
Suparna et al.,	0.03	0.02	0.04	< 0.0001	97
Desvarieux et al.,	0.03	0.02	0.05	<0.0001	97
Brand et al.,	0.03	0.02	0.05	< 0.0001	97
Parinello et al.,	0.03	0.02	0.05	< 0.0001	97

Cristofaro et al.,	0.03	0.01	0.04	<0.0001	96.9
Manfredi et al.,	0.03	0.012	0.04	< 0.0001	97
Calza et al.,	0.03	0.02	0.04	< 0.0001	97
van Vonderen et al.,	0.03	0.02	0.05	<0.0001	97
Seminari et al.,	0.03	0.01	0.04	< 0.0001	96.9

Table S3: Quality assessment of cohort studies using the Newcastle–Ottawa scale.

	Selection				Comparability	Exposure				
Study	Representativeness of Cohort	Selection of Non-Exposed Cohort	Ascertainment of Exposure	Outcome Not Present at Start	Comparability of Cohorts	Assessment of Outcome	Assessment of Outcome	Adequacy of Follow-Up	Total Score	Quality
Parrinello et al., 2012	★	★	★	★	★	★	★	★	8	High
Fourie et al., 2015	☆	★	★	★	★	★	★	★	7	High
Yacovo et al., 2020	★	★	★	★	★	★	★	★	8	High

Table S4: Quality assessment of case-control studies using the Newcastle–Ottawa scale.

	Selection				Comparability	Exposure				
Study	Case definition Adequate ?	Representativeness of the cases	Selection of controls	Definition of controls	Based on design and analysis	Ascertainment of exposure	Same method for cases and controls	Non-response rate	Total Score	Quality
Vohra & Bharti, 2017	★	★	★	★	★	★	★	☆	7	High
Van Vonderen et al., 2009	★	☆	★	★	★★	★	★	★	8	High

Table S5: Quality assessment of cross-sectional studies using the Newcastle–Ottawa scale.

	Selection				Comparability	Exposure			
Study	Representativeness of sample	Sample size	Nonrespondents	Ascertainment of exposure	Based on Design and Analysis	Assessment of outcome	Statistical test	Total Score	Quality
Joseph et al., 2025	★	★	★	★★	☆	★	★	7	High
Chikwati et al., 2024	★	★	☆	★★	★	★★	★	8	High
Sarfo et al., 2019	★	★	☆	★★	★	★★	★	8	High
Vos et al., 2019	★	★	☆	★★	★	★★	★	8	High
Gupta et al., 2018	★	☆	☆	★★	★	★★	★	7	High
Kirichenko et al., 2018	★	☆	☆	★★	★	★★	★	7	High
Sadiq et al., 2024	★	☆	★	★★	★	★★	★	8	High
Moshomo et al., 2024	★	☆	★	★★	★	★★	★	8	High
Gupta et al., 2022	★	☆	★	★★	☆	★	★	6	Moderate
Castro et al., 2021	★	☆	★	★	★	★	★	6	Moderate
Vos et al., 2020	★	★	☆	★	★	★★	★	7	High
Gleason et al., 2015	★	☆	☆	★★	★	★★	★	7	High
Goulenok et al., 2015	★	☆	☆	★★	★	★★	★	7	High
Pirs et al., 2014	★	☆	☆	★★	★	★★	★	7	High

Zhu et al., 2014	★	☆	☆	★★	★	★★	★	7	High
Desvarieux et al., 2013	★	★	★	★★	★	★★	★	9	High
Suparna et al., 2013	★	☆	★	★	★	★★	☆	6	Moderate
Manfredi et al., 2011	★	☆	★	★★	★	★★	★	8	High
Brand et al., 2011	★	☆	★	★★	★	★★	★	8	High
Cristofaro et al., 2011	★	☆	★	★★	★	★★	★	8	High
Calza et al., 2009	★	☆	★	★	★	★★	★	7	High
Seminari et al., 2002	★	☆	★	★	★	★★	★	7	High
